# A novel hypoxia- and lactate metabolism-related prognostic signature to characterize the immune landscape and predict immunotherapy response in osteosarcoma

**DOI:** 10.3389/fimmu.2024.1467052

**Published:** 2024-11-06

**Authors:** Yizhuo Wang, Xin Wang, Yang Liu, Jiayuan Xu, Jiyuan Zhu, Yufu Zheng, Quan Qi

**Affiliations:** ^1^ The Second Department of Orthopedic Surgery, First Affiliated Hospital of Harbin Medical University, Harbin, China; ^2^ The Fourth Department of Medical Oncology, Harbin Medical University Cancer Hospital, Harbin, China; ^3^ Department of pathology, First Affiliated Hospital of Harbin Medical University, Harbin, China

**Keywords:** hypoxia, lactate metabolism, osteosarcoma, gene signature, immune checkpoint inhibitors, immunotherapy, tumor microenvironment, single-cell sequencing

## Abstract

**Background:**

Immunotherapy has shown considerable promise in cancer treatment, yet only a minority of osteosarcoma patients derive benefits from this approach. Hypoxia and lactate metabolism are two predominant characteristics of the tumor microenvironment. These features are crucial for molding the immune landscape and thus have the potential to act as predictive indicators for immunotherapy response.

**Methods:**

Prognostic modeled genes were identified through univariate and multivariate Cox regression as well as LASSO regression analyses. The tumor microenvironment was evaluated using ESTIMATE, CIBERSORT, and ImmuCellAI analyses. Tide prediction and expression of immune checkpoints, MHC molecules, chemokines, interleukins, interferons, receptors, and other cytokines were utilized to estimate immunotherapy efficacy. Single-cell analysis was performed to demonstrate the expression of modeled genes among various immune cell types. Experimental validation was carried out to verify the expression and functions of *SFXN4* and *SQOR*.

**Results:**

A potent signature was constructed with 8 genes related to hypoxia and lactate metabolism, including *MAFF*, *COL5A2*, *FAM162A*, *SQOR*, *UQCRB*, *SFXN4*, *PFKFB2* and *COX6A2*. A nomogram incorporating risk scores and other clinical features demonstrated excellent predictive capacity. Osteosarcoma patients with high-risk scores exhibited poor prognosis and more “cold” tumor characteristics. According to the ESTIMATE algorithm, these patients displayed lower immune, stromal, and ESTIMATE scores, partially attributed to inadequate infiltration of key immunocytes. The Ciborsort analysis similarly indicated that high-risk individuals had diminished infiltration of critical anti-tumor immune cells such as Cytotoxic T cells, CD4+ T cells, and NK cells. The low expression levels of certain immune checkpoints, MHC molecules, chemokines, interleukins, interferons, receptors, and other cytokines in high-risk cases suggested their unsatisfactory responses to immune treatment. Tide prediction further demonstrated that fewer individuals classified as high risk may exhibit sensitivity to immune checkpoint inhibitor therapy. Notably, *SFXN4* was found to be highly expressed in osteosarcoma tissues and cells; it promoted the growth, migration, and invasion of osteosarcoma cells, while *SQOR* had the opposite effect.

**Conclusion:**

Our research has developed a robust hypoxia- and lactate metabolism-related gene signature, providing a solid theoretical foundation for prognosis prediction, classification of “cold” and “hot” tumors, accessing immunotherapy response, and directing personalized treatment for osteosarcoma.

## 1 Introduction

Osteosarcoma is a highly aggressive bone sarcoma of mesenchymal origin, predominantly occurring in young individuals and often leading to mortality as a result of lung metastases (1–3). The conventional therapeutic methods routinely applied in osteosarcoma, including neoadjuvant chemotherapy, surgical resection, and postoperative adjuvant chemotherapy, have remained largely unchanged for many years (4–7). Compared to patients with local diseases whose survival rate at five years reached 65-70% following these treatments, those with metastatic disease had a significantly lower survival rate of only 20% (8–10). Hence, it is urgent to identify reliable tools for accurate initial diagnosis, introduce novel therapeutic strategies for osteosarcoma, and establish methods for ongoing monitoring of treatment efficacy (11).

Hypoxia signifies a reduction in the tissue partial pressure of oxygen [ptiO (2)], which is a critical characteristic of the tumor microenvironment (TME) commonly observed in multiple solid tumors (12). Any factor triggering an imbalance between the high proliferative capacity of cancer cells and oxygen availability, such as overlong diffusion distances and structural abnormalities in vascular vessels, can contribute to hypoxia (13). Notably, tumors can adapt to hypoxic conditions to promote growth, aggressiveness, angiogenesis, metastatic potential, and resistance to treatment through transcription factors, especially hypoxia-inducible factor 1α (HIF1α) (14). Given the impact of hypoxia on the malignant biological function of osteosarcoma, identifying key signature genes within the hypoxia gene set could facilitate the development of therapies targeting hypoxia-induced tumorigenesis in osteosarcoma.

Lactate is more than a mere metabolite and also acts as a signaling molecule whose intracellular and extracellular concentrations rise because of the essential effect of aerobic glycolysis in energy production (15, 16). Hypoxia invariably triggers lactate production, which subsequently accumulates in the TME, in turn facilitating hypoxic response (17). In addition, Zhang et al. has identified lactylation as a significant epigenetic modification modulating the transcription of numerous oncogenes and tumor suppressor genes, thereby influencing tumor progression (18). Several studies have highlighted the impact of lactic acid on tumor cell progression, metastasis, epithelial-mesenchymal transition, angiogenesis, and immune evasion (19–21). Considering the multifaceted roles of lactate, a comprehensive understanding of the mechanisms by which lactate metabolism-related genes operate in osteosarcoma will supply valuable insights into tumor reprogramming and therapeutic strategies.

Metabolic disorders in cancers are marked by hypoxia and subsequent elevated levels of lactate (22). This interplay contributes to the creation of a restrictive TME dampening the efficacy of immune effector cells, and ultimately weakens the immune system’s ability to combat tumors (23). Hypoxia plays a vital role in promoting tumor immune evasion by upregulating certain immunosuppressive immune checkpoints, cytokines, growth factors, and interleukins to accelerate tumor progression (24). Furthermore, hypoxia hinders immune response by reducing infiltration and function of CD8+ T cells, impairing maturation and activities of dendritic cells coupled with natural killer cells, evoking M2-like polarization of tumor-associated macrophages, and boosting regulatory T cells and myeloid-derived suppressor cells activities (25, 26). Simultaneously, lactate augments antitumor immunity by driving M2 polarization, enhancing stemness of CD8+ T cells, and promoting PD-1 expression in regulatory T cells (27–29).

In this study, our objective was to comprehensively elucidate the heterogeneity of the TME and precisely predict the effects of immunotherapy in osteosarcoma. In terms of the impact of hypoxia and lactate metabolism on tumor immunity, we identified 8 genes related to hypoxia and lactate metabolism as potential predictive markers and developed a hypoxia- and lactate metabolism-related gene signature that held promise for clinical application. Osteosarcoma patients stratified based on this prognostic model exhibited distinct survival outcomes, TME characteristics, immune cell infiltration, expression levels of immune checkpoints, immune response, and sensitivity to drugs.

## 2 Materials and methods

### 2.1 Data source and preprocessing

Transcriptional expression data and corresponding clinical information of osteosarcoma samples were sourced from the TARGET (https://ocg.cancer.gov/programs/target) database and GEO (http://www.ncbi.nlm.nih.gov/geo/) database. Transcriptome data of para-cancerous tissues were retrieved from the GTEx (https://xenabrowser.net/datapages/) database. Hypoxia-related genes (HRGs) and lactate metabolism-related genes (LMRGs) were obtained from The Molecular Signatures Database (MSigDB, https://www.gsea.msigdb.org/gsea/msigdb/index.jsp). By exploring the keywords “hypoxia” and “lactate”, we found 5 priority LMRG datasets and 7 major HRG datasets in the MSigDB database. After filtering out duplicate genes in the identified LMRG and HRG datasets, a total of 284 LMRGs and 493 HRGs were selected for the forthcoming study (**Supplementary Table S1**).

### 2.2 Identification of DEGs, DELMRGs and DEHRGs

The R package “limma” was utilized to screen differentially expressed genes (DEGs) meeting the criteria of absolute fold change greater than 2 and *p* values less than 0.05. Differentially expressed hypoxia-related genes (DEHRGs) were determined by intersecting DEGs with HRGs, while differentially expressed lactate metabolism-related genes (DELMRGs) were recognized by intersecting DEGs with LMRGs.

### 2.3 Development of a hypoxia- and lactate metabolism-related gene signature

Univariate Cox regression analysis was conducted based on the R package “survival” to pick out meaningful candidate genes associated with the overall survival time of patients among DELMRGs and DEHRGs. Genes were deemed statistically significant if their *p* values were less than 0.05. To mitigate overfitting and refine gene selection, the least absolute shrinkage and selection operator (LASSO) Cox regression analysis was performed. Finally, a total of 8 genes were chosen for inclusion in the gene signature based on multivariate Cox regression analysis.

### 2.4 Derivation of the prognosis classifier

The following equation was used to determine the risk score: risk score = (0.6049967×*UQCRB*) + (-0.4744143×*SQOR*) + (0.4064687×*COL5A2*) + (0.5114636×*FAM162A*) + (0.8507392×*SFXN4*) + (-1.187998×*PFKFB2*) + (0.4085068×*MAFF*) + (0.2279495×*COX6A2*). Subsequently, the risk score of each osteosarcoma sample in the three datasets were calculated. In accordance with the median risk score of each database, the osteosarcoma samples were then stratified into two groups: low-risk and high-risk.

### 2.5 Survival analysis and measurement of predictive ability

The R package “survminer” was utilized for conducting Kaplan-Meier survival analysis to compare survival between the two risk groups using the optimal cutoff value of gene expression. The prediction performance of this model was appraised by employing the “timeROC” package to generate receiver operating characteristic (ROC) curves and compute the corresponding area under the curve (AUC) values.

### 2.6 Construction of the prognostic nomogram

A nomogram was organized to predict the 1-, 3-, and 5-year survival rates of osteosarcoma patients. The predictive accuracy of the nomogram was rated using ROC curves, C-index, and calibration curves. The net benefit of the nomogram and other clinical characteristics was assessed by Decision curve analysis (DCA).

### 2.7 Functional annotation enrichment

The R package “clusterProfiler” was utilized for conducting GO functional enrichment analysis, while the DAVID (https://david.ncifcrf.gov/summary.jsp) online database was searched for KEGG functional enrichment analysis. The top 10 results from the GO functional enrichment analysis in terms of 3 distinct aspects were selected based on smaller *p* values. Similarly, the top 10 results from the KEGG analysis were also chosen. Bar charts and bubble charts were visualized using the “ggplot2” package.

### 2.8 Evaluation of tumor microenvironment pathways

The 29 pathways related to the tumor microenvironment (TME) were initially identified in a previous study (30). Subsequently, the R package “clusterProfiler” was used to calculate single-sample gene set enrichment analysis (ssGSEA) scores to quantify the pathway activity. Then, we identified statistically significant associations among the various TME pathways. The “ggcor” package was utilized to unmask the correlation between risk scores and the TME.

### 2.9 Immune status analyses

The immune cell abundance identifier (ImmuCellAI) was employed to evaluate the infiltration levels of 24 immune cell types in the TARGET database and GSE21257 dataset. Additionally, the CIBERSORT algorithm was conducted to appraise the infiltration abundance of 22 immune cell types. The correlation analysis was for investigating the relationship between the expression of the 8 HLMRGs and cell infiltration inferred from ImmuCellAI and CIBERSORT algorithm. Moreover, four key indicators of the tumor microenvironment components were evaluated through the “estimate” R package.

### 2.10 Immunotherapy and molecular therapy response prediction

Chemokines, interleukins, interferons, receptors, and other cytokines were sourced from a published article (31). The TIDE online tool was employed to forecast potential clinical response to immunotherapy in osteosarcoma patients. A higher TIDE prediction score typically indicates increased immune evasion by the tumor and reduced likelihood of positive response to immune checkpoint inhibitors (ICIs). Genes associated with T-cell-inflamed gene expression profile (GEP), Th1/IFNγ gene signature, and cytolytic activity were obtained from a prior study and their scores were computed using the “ssGSEA” algorithm to predict response to immune checkpoint therapy (30).

### 2.11 Drug sensitivity analysis

The diversity in medication sensitivity was investigated by conducting a comparison of the IC50 values of more than 100 chemotherapeutic agents through the R package “pRRophetic”. Setting a significance threshold of *p* smaller than 0.005, we identified several efficacious chemotherapy agents in both risk groups.

### 2.12 Single-cell analysis

The scRNA sequence matrix was imported using the R package “Seurat.” To acquire high-quality scRNA-seq data, specific filtering conditions were applied for every cell: only cells with over 500 UMI counts were included; cells expressing fewer than 300 genes and equal to or more than 5000 genes were excluded; cells with over 10% mitochondrial gene expression were also eliminated. Normalization of the scRNA-seq data was carried out by applying the “NormalizeData” function, with the normalization method set to “LogNormalize”. The top 3,000 highly variable genes were recognized through the “FindVariableFeatures” function. Subsequently, principal component analysis (PCA) was carried out to lessen the dimensionality of the raw data based on the top 3,000 genes using the “RunPCA” function. Principal components with statistical significance were discerned using “JackStraw” function, and the top 14 meaningful principal components were selected for cell clustering based on variance explained. Cell clustering analysis was conducted using the “FindNeighbors” and “FindClusters” functions, followed by uniform manifold approximation and projection (UMAP) analysis through the “RunUMAP” function. Batch correction was implemented by applying the “Harmony” package. The “FindAllMarkers” function was carried out to identify marker genes in each cluster. The cell annotation was from a previously published paper (32).

### 2.13 Cell culture

Human osteosarcoma cell lines (HOS, 143B) and human normal osteoblast cell lines (hFOB1.19) were purchased from Procell Life Science and Technology Co., Ltd. (Wuhan, China). Cells were cultured with 10% fetal bovine serum (PAN, German) and 1% penicillin-streptomycin solution (SEVEN, China) in a humidified environment at 5% CO2 and 37°C.

### 2.14 Transfections

Tumor cells were cultured in a six-well plate at a density of 3×10^5^ cells per well in appropriate culture medium and incubated overnight with agitation. Upon reaching a cell density of 60 to 70%, the cells were washed with PBS and supplemented with serum-free culture medium, followed by the addition of the plasmid-lipo2000 mixture to each well. The six-well plates were then incubated in the incubator for 6 hours before being switched to serum-containing culture medium. The sequence of the siRNA purchased from General Biol (China) is as follows:GAUCAAAGCUAGAGUGACUTT (si-*SQOR*-1); ATCTTTACCTTCCCAAATACTCC (si-*SQOR*-2); GGACAAAGGCUGUUAGAGATT (si-*SFXN4*-1); CUGACUGGCCCUUGGAUUATT (si-*SFXN4*-2).

For virus transfection, 143B cells were seeded in 24-well plates at a density of 1 × 10^5^ cells per well and incubated for 18 to 24 hours. The original medium was replaced with 2 ml of fresh medium containing 6 µg/ml polybrene, along with an appropriate quantity of virus (Genechem, Shanghai, China). After four hours, an additional 2 ml of fresh medium was added to dilute the polybrene. Forty-eight hours post-transfection, the cells were screened using new medium supplemented with puromycin. Following another forty-eight hours, the medium was again replaced with fresh medium before harvesting the cells.

### 2.15 CCK8 assay

100 μL of suspension containing 5000 transfected cells was dispensed into each well of a 96-well plate along with 10 μL of CCK8 solution (Beyotime, China). The plate was then placed in a cell culture incubator for 1 hour, following which absorbance readings were taken at 450 nm and recorded.

### 2.16 Cell colony assay

3600 osteosarcoma cells were equally distributed into six-well plates and incubated at 37°C with 5% CO2 for 14 days with regular medium changes. Post-incubation, the cells were fixed and stained using 4% paraformaldehyde and 0.1% crystal violet for 20 minutes each, after which images were captured and data documented.

### 2.17 Scratch assay

Upon the cell density reaching 80 to 90 percent on a six-well plate, a scratch was created using a pipette tip perpendicular to the plate surface. Following a wash with PBS to remove unattached cells, microscopic images were captured at 0 and 48 hours.

### 2.18 Transwell assay

The transfected HOS and 143B cell lines were cultured with the serum-free DMEM and serum-free 1640, respectively, in a Transwell upper chamber. Corresponding culture medium containing 10% FBS was added to the lower chamber. The cells were incubated at 37°C with 5% CO2 for 48 hours, fixed with formaldehyde, stained with crystal violet, and visualized under a microscope for analysis.

### 2.19 qRT-PCR

The total RNA was extracted from HOS and 143B as well as hFOB1.19 using TRIzol reagent (Invitrogen, USA). Following the manufacturer’s instructions, the PrimeScriptTM RT reagent kit (Takara, Japan) was used to create the cDNA by reverse transcription. The relative expression of *SQOR* and *SFXN4* was measured according to the 2-ΔΔCt algorithm by normalizing the samples to GAPDH utilizing a LightCycler 480 Fluorescence Quantification System (Roche, Basel, Switzerland).

### 2.20 Western blotting

The Radioimmunoprecipitation (RIPA) lysis buffer (Beyotime, China) was used to isolate the proteins and then transferred the proteins to onto PVDF membranes (Millipore, Billerica, MA, USA) by electrophoretic separation. The membranes were closed for one hour at room temperature with 5% nonfat milk in TBST before being incubated at 4° with a particular primary antibody. After overnight incubation the bands were visualized using the ECL kit. Specific antibodies included: *SFXN4* (YT4298, Immunoway Biotechnology Company, China), *SQOR* (21112-1-AP, Proteintech Group Inc., Wuhan, China).

### 2.21 Tissue specimens and immunohistochemistry

The expression of two genes (*SQOR* and *SFXN4*) significantly affecting prognosis was confirmed through immunohistochemical experiments. Four osteosarcoma patients from the First Affiliated Hospital of Harbin Medical University voluntarily donated tumor tissues and normal bone tissues. All of them signed informed consent forms before tissue donation. Prior to surgery, none of them received radiation therapy or chemotherapy. The hospital ethics committee gave approval for this research. Slices of tissues were fixed, dehydrated, and paraffin-embedded. Then, the tissues were dewaxed and hydrated through rinses with xylene, anhydrous ethanol, ethanol, and distilled water after baking at 60°C. For 12 minutes, tissues were submerged in 0.3% H_2_O_2_ to deactivate endogenous peroxidase. Sections were sealed with serum and then treated with the appropriate antibodies for an overnight period at 4°C. Next, all sections were exposed to secondary antibodies for 30 minutes at 37°C. Two pathologists employed 3,3-diaminobenzidine (DAB) reagent (Boster, Wuhan, China) to stain every tissue piece. The antibodies utilized in the aforementioned *SQOR* (1:200) and *SFXN4* (1:200) experiments were both from. Microscopic images were captured and analyzed using cellSns software.

### 2.22 *In vivo* experiment

The BALB/c nude mice were procured from Huachuang Sino (Jiangsu, China) and housed at the Animal Experiment Center of the First Hospital of Harbin Medical University. A total of ten four-week-old nude mice were randomly assigned to two groups and subcutaneously injected bilaterally with 1×10^6^ cells each of 143B wild-type, *SFXN4* knockdown, and *SQOR* knockdown cells. Tumor volume was monitored and measured continuously for a period of 15 days post-implantation. Following this observation period, the mice were euthanized. All procedures involving the nude mice adhered strictly to the regulations set forth by the Ethics Committee of the First Hospital of Harbin Medical University (Batch Number: IRB-AF/SC-0402.0).

### 2.23 Statistical analysis

The Wilcoxon signed-rank test was applied to recognize differences between two groups. Spearman’s rank correlation coefficient was employed to assess the correlation between risk scores and pathway enrichment scores calculated by the “ssGSEA” algorithm. A *p* value less than 0.05 was considered as statistically significant. * denotes *p* < 0.05, ** represents *p* < 0.01, *** means *p* < 0.001 and **** indicates *p* < 0.0001.

## 3 Results

### 3.1 Construction of the prognostic HLMRGS and validation of its predictive ability

**Figure 1:**
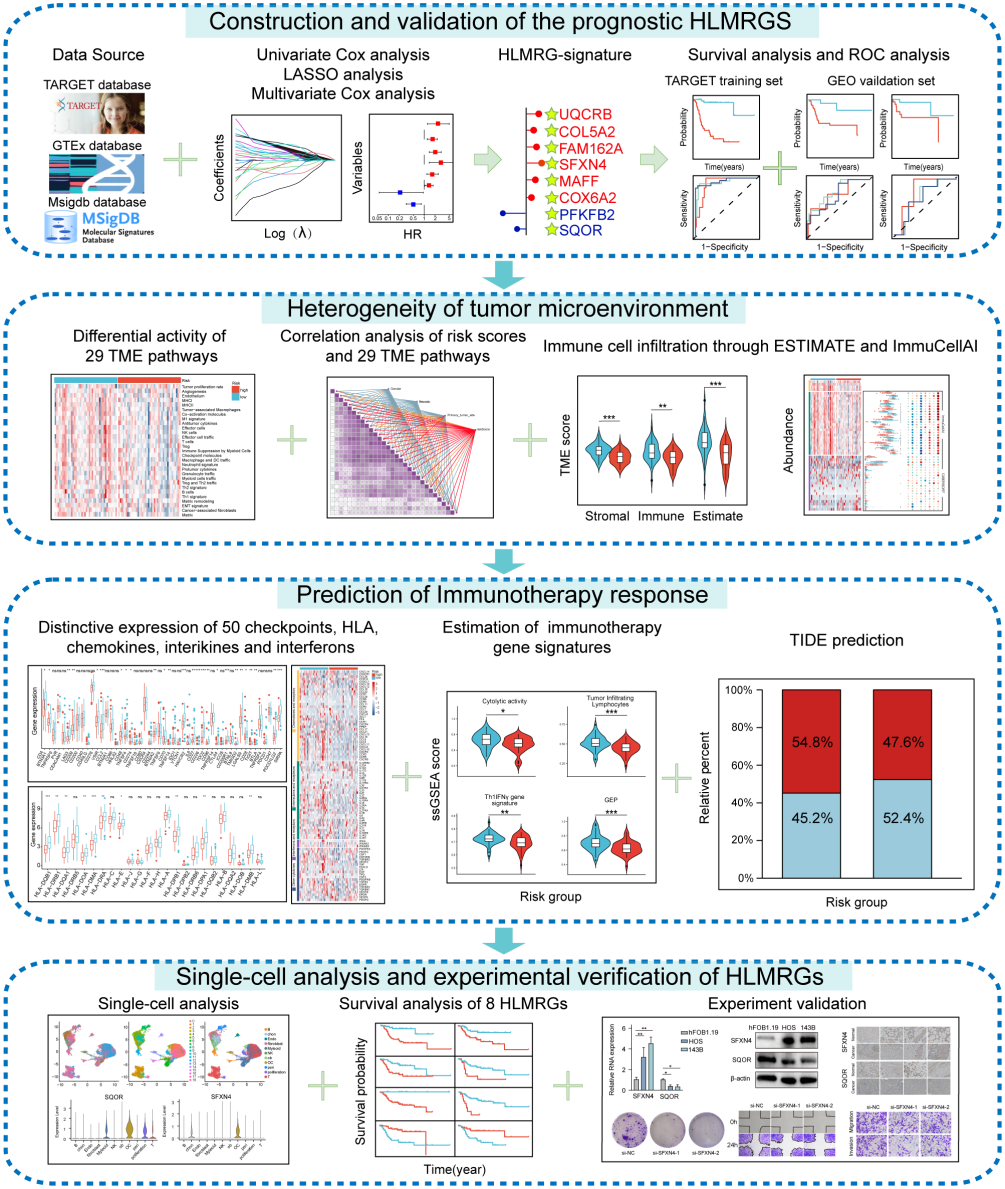
Flow chart of this study.

**Figure 2:**
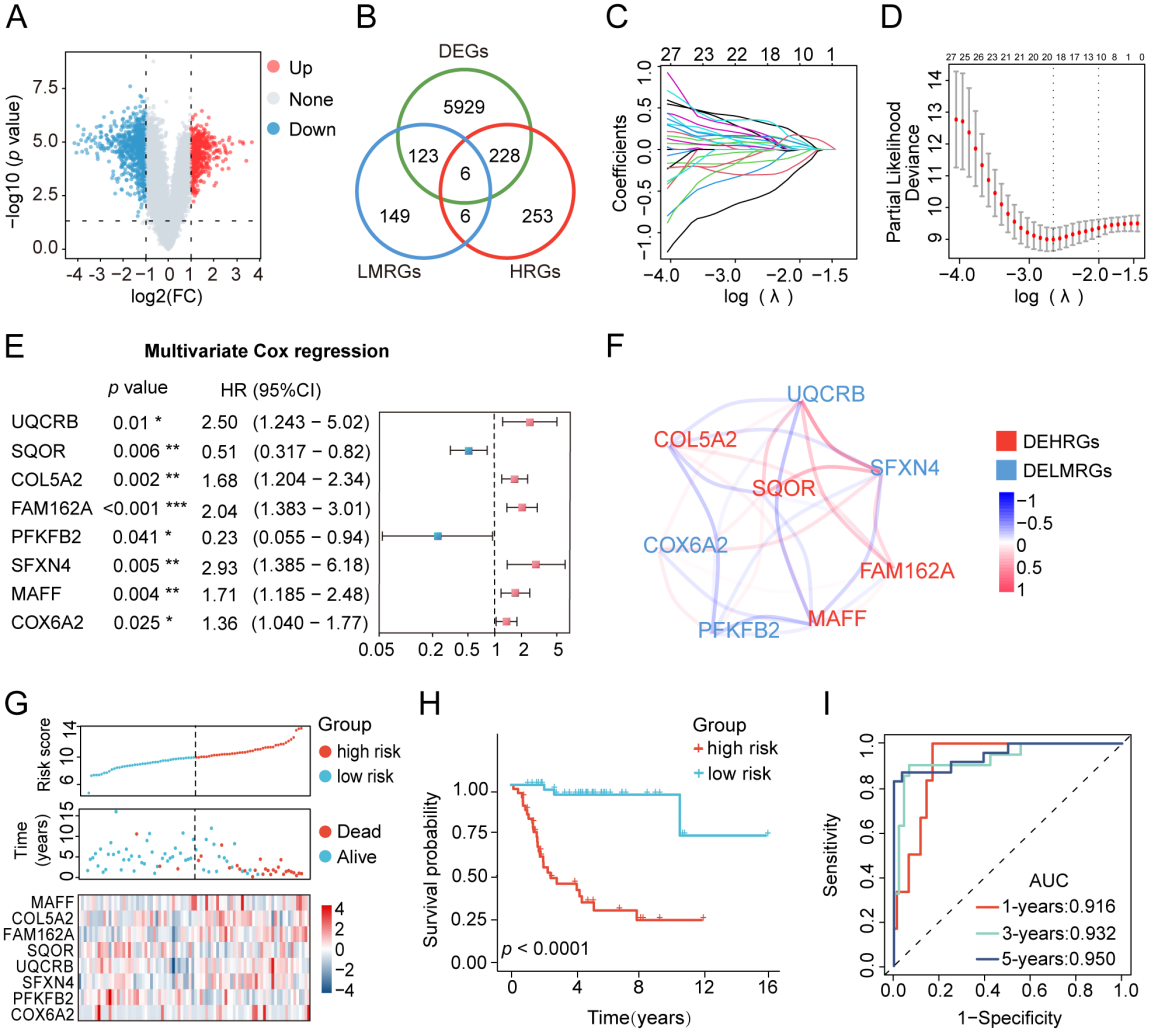
Development of the HLMRGS and verification of its predictive ability in the TARGET database. **(A)** Volcano chart of 6286 DEGs between osteosarcoma and normal samples. **(B)** Venn diagram depicting the junction of HRGs, LRGs, and DEGs. **(C, D)** LASSO penalized Cox regression, and **(E)** multivariate Cox regression analyses of the DEHRGs and DELMRGs. **(F)** The association network diagram and types of 8 HLMRGs. **(G)** The risk scores, clinical outcomes, and expression of 8 modeled genes of osteosarcoma samples divided by median risk score. **(H)** KM survival analysis for the two risk cohorts. **(I)** The ROC curve of HLMRGS at 1, 3, 5 years.

The research process was illustrated in **Figure 1**. The differential analysis of transcriptome data between osteosarcoma patients in the TARGET database and normal specimens in the GTEx database revealed 2925 upregulated genes and 3361 downregulated genes (**Figure 2A**). A Venn diagram depicted 234 differentially expressed hypoxia-related genes (DEHRGs) and 129 differentially expressed lactate metabolism-related genes (DELMRGs), with 6 shared genes between them (**Figure 2B**). Univariate Cox regression analysis identified 51 genes out of the DEHRGs and DELMRGs that were associated with prognosis, including 20 DEHRGs and 31 DELMRGs (**Supplementary Table S2**). Subsequent LASSO Cox regression analysis was carried out to reduce overfitting and filtered 8 genes (**Figures 2C, D**). Multivariate Cox regression analysis confirmed that these 8 identified genes, including 2 protective elements (*SQOR* and *PFKFB2*) and 6 hazardous factors (*MAFF*, *COL5A2*, *FAM162A*, *UQCRB*, *SFXN4*, and *COX6A2*), could independently predict the overall survival of osteosarcoma samples (**Figure 2E**). Eventually, an optimal prognostic gene signature named hypoxia- and lactate metabolism-related gene signature (HLMRGS) was generated with those 8 determined genes. The correlation of 8 genes was portrayed as follows (**Figure 2F**). Among these genes, *MAFF*, *COL5A2*, *FAM162A* and *SQOR* were DEHRGs, while *UQCRB*, *SFXN4*, *PFKFB2* and *COX6A2* were DELMRGs. According to the Kaplan-Meier (KM) survival analysis of 8 modeled genes, elevated expression levels of *COL5A2*, *COX6A2*, *UQCRB*, *SFXN4*, *FAM162A* and *MAFF* sharply shortened the survival time of osteosarcoma patients (**Supplementary Figures S1A–H**). Conversely, high expression levels of *PFKFB2* and *SQOR* were associated with prolonged survival. The risk scores derived from the expression levels of these 8 modeled genes effectively categorized samples into low- and high-risk groups. The findings indicated that high-risk patients experienced worse clinical outcomes (**Figures 2G, H**). Receiver operating characteristic (ROC) analysis reflecting the sensitivity and specificity of gene signatures showed that the area under curve (AUC) values of the HLMRGS were 0.916 at 1 year, 0.932 at 3 years and 0.950 at 5 years (**Figure 2I**). After the establishment of HLMRGS within the TARGET database, its prognostic significance and predictive capacity were verified externally in the GSE39055 and GSE21257 datasets, thereby affirming both its repeatability and reliability (**Supplementary Figures S2A–F**). Altogether, the collective findings unequivocally validated the prognostic value of the HLMRGS and its predictive potential.

### 3.2 Stratified analysis by different clinical characteristics of osteosarcoma patients

To further elucidate the universal applicability of the HLMRGS, we conducted survival outcome predictions for osteosarcoma patients stratified by various clinical characteristics using the HLMRGS. Osteosarcoma samples from the TARGET database were grouped into diverse subcategories bottomed on age (≥18 *vs.* <18 years), gender (male *vs.* female), metastatic stage (metastatic *vs.* nonmetastatic), primary tumor location (leg *vs.* arm/pelvis), and specific tumor site (lower limb *vs.* upper limb *vs.* pelvis/sacrum/ilium) (**Supplementary Table S3**). KM survival analysis was performed across these subgroups, illustrating that individuals with high-risk scores exhibited significantly poorer outcomes amidst most subgroups, including age, gender, and metastatic stage (**Supplementary Figures S3A–K**).

### 3.3 Clinical value of the HLMRGS in osteosarcoma

**Figure 3:**
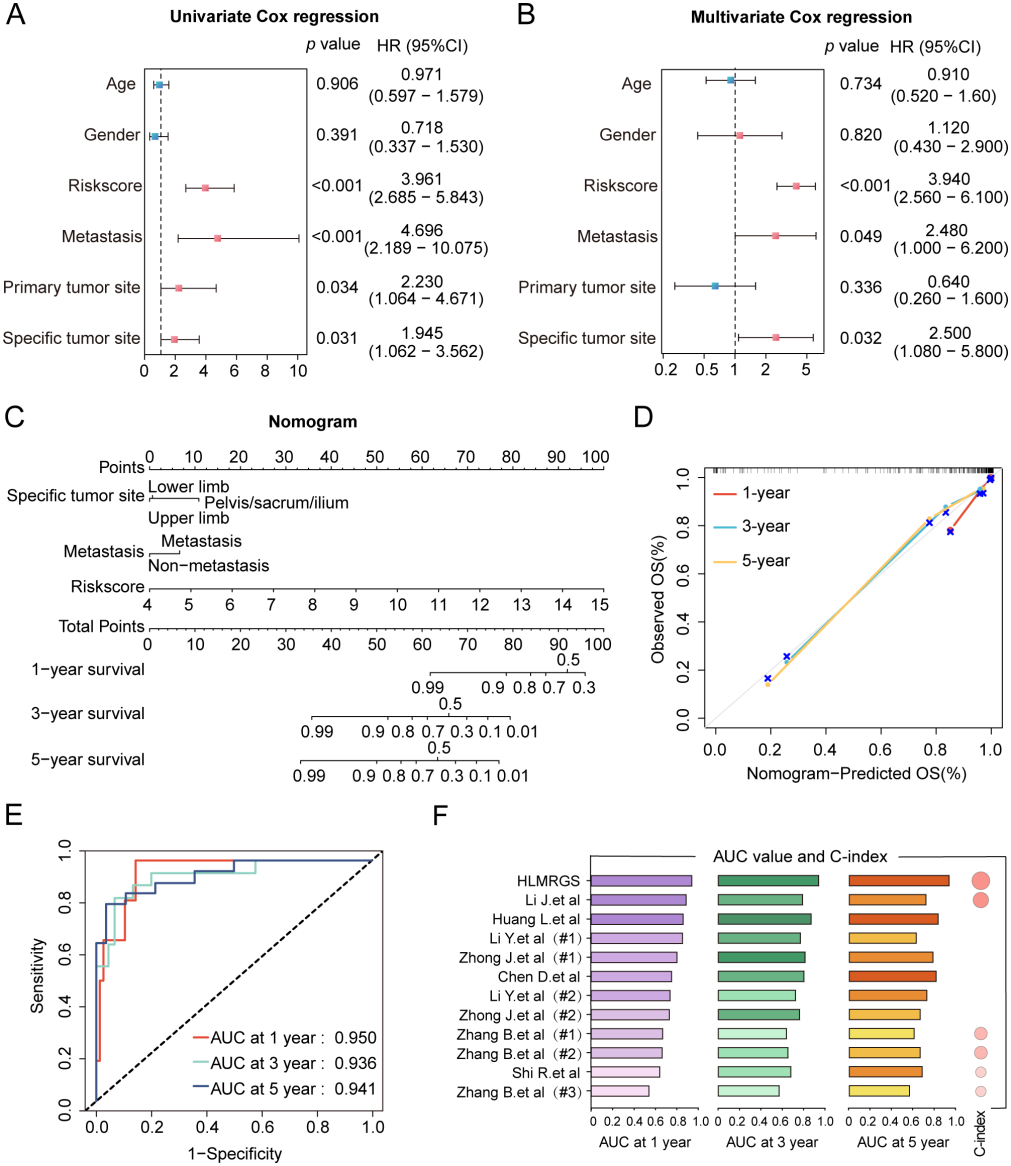
Construction of the clinical nomogram and validation of its predictive power in the TARGET database. **(A, B)** The forest diagram of univariate and multivariate Cox regression analyses comprising risk scores and distinctive clinical features. **(C)** The nomogram based on three factors with statistical significance, involving metastasis, specific tumor site and risk scores. Total scores were summed up by points of each variable. The survival probabilities in three different time periods are indicated on the axis at the bottom. **(D)** Calibration curves showing a tremendous fit between the predictive outcomes and actual outcomes. **(E)** ROC curves of the nomogram depicting the prognostic performance of HLMRGS. **(F)** Comparison of AUC values and C-index between the HLMRGS and other gene signatures related with hypoxia or lactate metabolism.

After conducting univariate and multivariate Cox regression analyses, a nomogram was developed to validate the clinical significance of the HLMRGS. The univariate Cox regression analysis indicated that metastasis, primary tumor site, specific tumor site, and risk scores were relevant to overall survival time of osteosarcoma specimens from the TARGET database (**Figure 3A**). In addition, the multivariate Cox proportional hazard models revealed that metastasis, specific tumor site, and risk scores were independent prognostic factors for osteosarcoma patients (**Figure 3B**). Then, a nomogram model was structured based on these three independent prognostic indicators to predict the 1-, 3-, and 5-year survival probabilities of osteosarcoma samples (**Figure 3C**). The calibration plots depicted a satisfactory agreement between predicted and actual outcomes with a high C-index of 0.915 (**Figure 3D**). The AUC values of the nomogram were a whopping 0.916 at 1 year, 0.932 at 3 years and 0.950 at 5 years (**Figure 3E**). The decision curve analysis (DCA) curve suggested that the nomogram provided the highest net benefit, strongly confirming its clinical suitability in osteosarcoma patients (**Supplementary Figure S3L**). In particular, the HLMRGS displayed superior accuracy in comparison to previously published gene signatures related with hypoxia or lactate metabolism, as evidenced by its highest AUC value and C-index (**Figure 3F**). Overall, these results highlighted the applicability of the HLMRGS in clinical practice for osteosarcoma patients.

### 3.4 Functional enrichment analysis based on the HLMRGS

To explore the functional enrichment disparities between the two cohorts delineated by the HLMRGS, we conducted GO and KEGG enrichment analyses based on the differentially expressed genes (DEGs) between the low- and high-risk groups in the TARGET database. A total of 84 DEGs were discerned between these two groups, with 47 genes highly expressed and 37 genes downregulated (**Supplementary Figure S4A**). KEGG pathway analysis revealed evident enrichment of DEGs in pyrimidine metabolism, drug metabolism, EGFR tyrosine kinase inhibitor resistance and TGF-β signaling pathways (**Supplementary Figure S4B**). Additionally, GO pathway analysis exhibited predominant enrichment in the regulation of cellular response to growth factor stimulus, immunoglobulin complex, and signaling receptor activator activity (**Supplementary Figure S4C**).

### 3.5 Heterogeneity of the TME between the two risk groups

**Figure 4:**
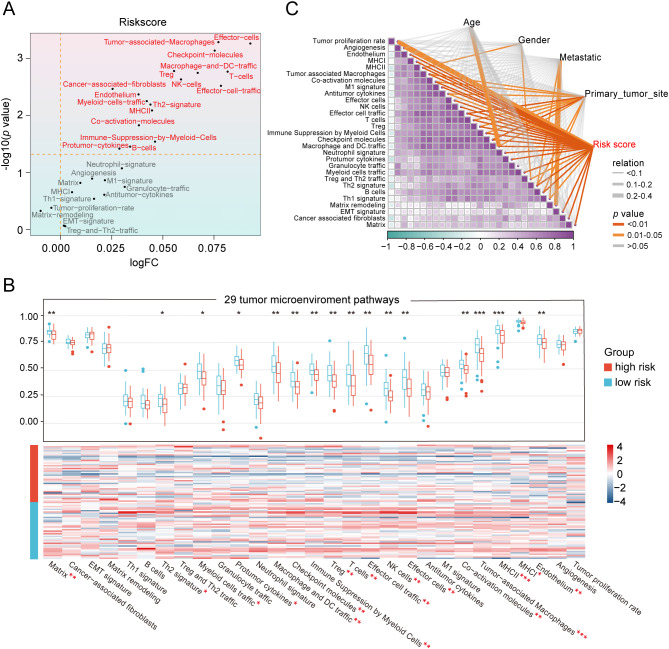
Heterogeneity of the tumor microenvironment between the two risk groups in the TARGET database. **(A)** The differentially expressed activity of 29 TME pathways between the two subgroups using “limma” package. **(B)** Heatmap and box line plot depicting the activity discrepancies of 29 TME pathways between the two cohorts. **(C)** Multidimensional correlation plot demonstrating the relationship of 29 TME pathways, and risk score as well as other clinical features. ∗ *p* < 0.05, ∗∗ *p* < 0.01, ∗∗∗ *p* < 0.001.

To unravel the heterogeneity within the TME, we did single-sample gene set enrichment analysis (ssGSEA) to estimate the activity of 29 TME pathways encompassing immune, matrix, and biological characteristics. Osteosarcoma patients were stratified into two cohorts according to their risk scores and distinct clinical features to compare the activity of 29 TME pathways. The two subgroups separated by only five factors namely, sex, age, metastasis, tumor site, and risk scores, demonstrated differences in TME pathway activities (**Figure 4A** and **Supplementary Figure S5A**). Notably, the risk scores-based group showed the most significant disparities in the activity of a total of 17 TME pathways compared to other groups. In addition, only 11 TME pathways linked with immunity exhibited obvious differences when categorized by risk scores (**Figure 4B** and **Supplementary Figure S5B**). These pathways reflected elevated activity levels in the low-risk group, as observed in both the TARGET database and the GSE21257 dataset. The pathways identified included MHC-II, coactivation molecules, checkpoint molecules, effector cells, NK cells, T cells, cancer-associated fibroblasts, myeloid cell traffic, macrophage and DC traffic, effector cell traffic and Th2 signature. More importantly, the risk scores displayed the strongest correlation with all 29 TME pathways, with a high degree of interrelation among the majority of TME gene signatures (**Figure 4C** and **Supplementary Figure S5C**).

**Figure 5:**
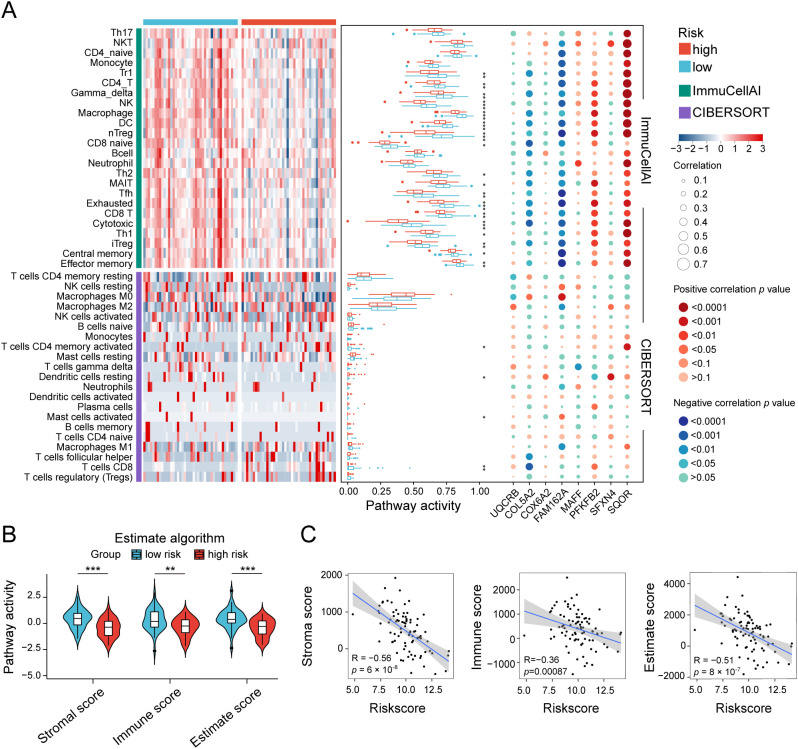
Divergences in the immune landscape between the low- and high-risk groups in the TARGET dataset. **(A)** Heatmap and box line plot showing the variances in immune infiltration with the ImmuCellAI and CIBERSORT algorithms. Bubble diagram depicting the correlation between the modeled genes expression and ImmuCellAI, and CIBERSORT scores. **(B)** Comparison of stromal, immune, and ESTIMATE scores between the two groups. **(C)** Correlation analysis of risk scores with stromal, immune, and ESTIMATE scores. ∗ *p* < 0.05, ∗∗ *p* < 0.01, ∗∗∗ *p* < 0.001.

Since the risk scores were primarily associated with immunity, we aimed to quantify the infiltration of diverse immune cell types to further elucidate the heterogeneity of the TME by using three different methodologies: ImmuCellAI, Cibersort and ESTIMATE (**Figure 5A** and **Supplementary Figure S6A**). Among the 8 key genes comprising the HLMRG, the protective factors *SQOR* and *PFKFB2* exhibited positive correlations with the abundance of most infiltrating immune cells, while the risk factors *FAM162A* and *COL5A2* showed negative associations with immune cell infiltration in both the TARGET and GSE21257 cohorts (**Figure 5A** and **Supplementary Figure S6B**). In accordance with the ImmuCellAI database, low-risk patients had a noticeably enriched population of some immune-activated cell types in both the TARGET and GSE21257 cohorts, such as CD4+ T cells, CD8+ T cells, cytotoxic T cells, dendritic cells and natural killer cells, manifesting the latent capacity of humoral immunity and cellular immunity in low-risk patients (**Figure 5A** and **Supplementary Figure S6C**). Regrettably, the distribution differences of most immune cells between the two risk groups obtained by the CIBERSORT algorithm were not statistically significant in both the TARGET and GSE21257 cohorts (**Figure 5A** and **Supplementary Figure S6D**). ESTIMATE analysis revealed that the low-risk group displayed increased stromal, immune, and ESTIMATE scores in the TARGET database (**Figure 5B**). The results were consistent in the GSE21257 cohort, with the exception of stromal scores, where statistical significance was not observed (**Supplementary Figure S6E**). Furthermore, the risk scores were inversely correlated with stromal, immune, and ESTIMATE scores in both the TARGET and GSE21257 cohorts (**Figure 5C** and **Supplementary Figure S6F**). In brief, the osteosarcoma patients with low-risk scores exhibited more “hot” tumor features. These results suggested that the two groups classified by the HLMRGS displayed quite different infiltration of certain key immune cells, which may be one of the explanations for the distinction in survival outcomes between them.

### 3.6 Prediction of response to immunotherapy and chemotherapy

**Figure 6:**
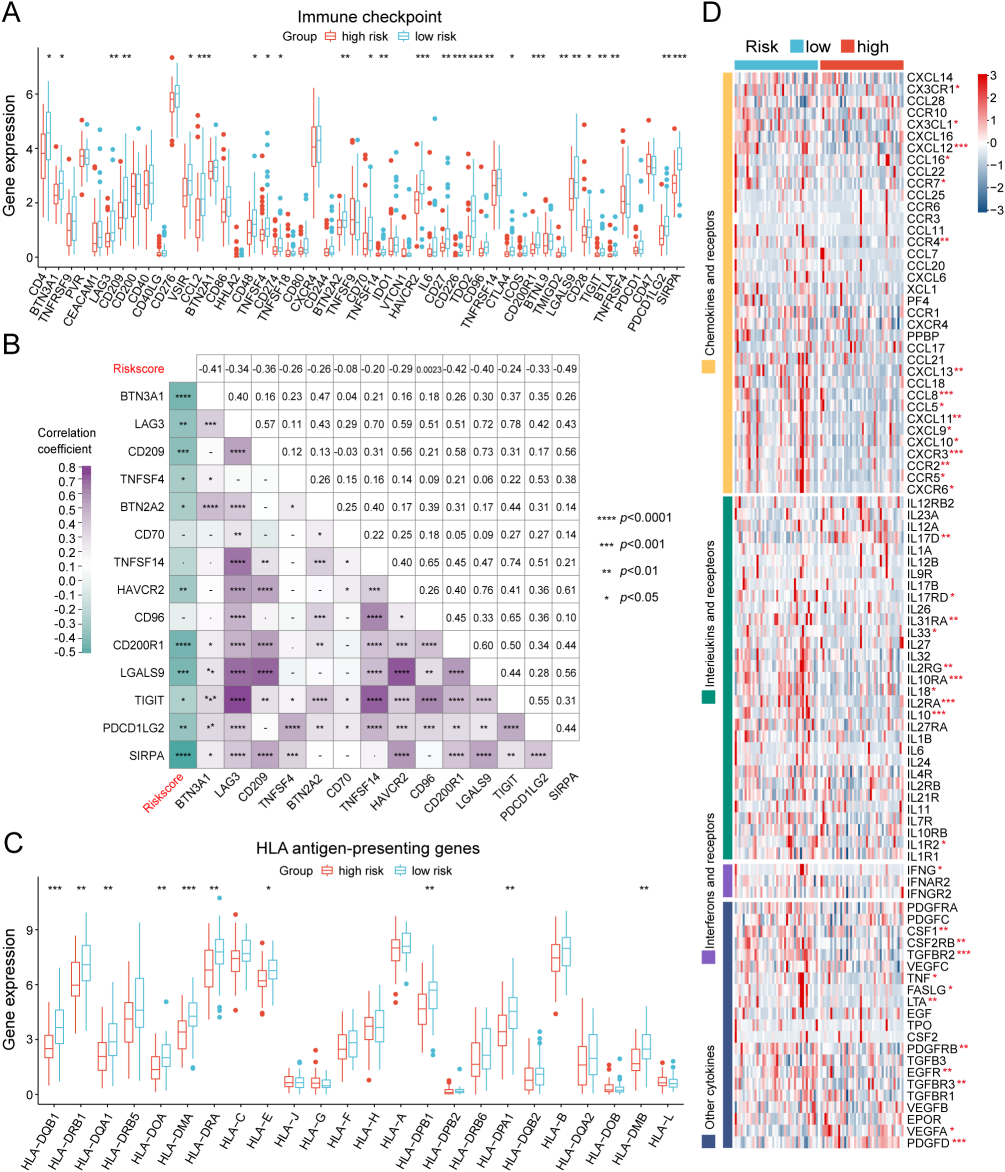
Evaluation of immunotherapy response of the low- and high-risk groups in the TARGET database. **(A)** Box plot showing the expression variations in immune checkpoints between the two cohorts. **(B)** Multidimensional correlation plot illustrating the relationship of risk scores and the gene expression of immune checkpoints reaching statistically significant differences in both the TARGET and GSE21257 cohorts. **(C)** Expression divergences of MHC molecules of the two subgroups. **(D)** Heatmap illustrating the differences in the gene expression of chemokines, interleukins, interferons, receptors, and other cytokines between the two risk groups. ∗ *p* < 0.05, ∗∗ *p* < 0.01, ∗∗∗ *p* < 0.001, ∗∗∗∗ *p* < 0.0001.

**Figure 7:**
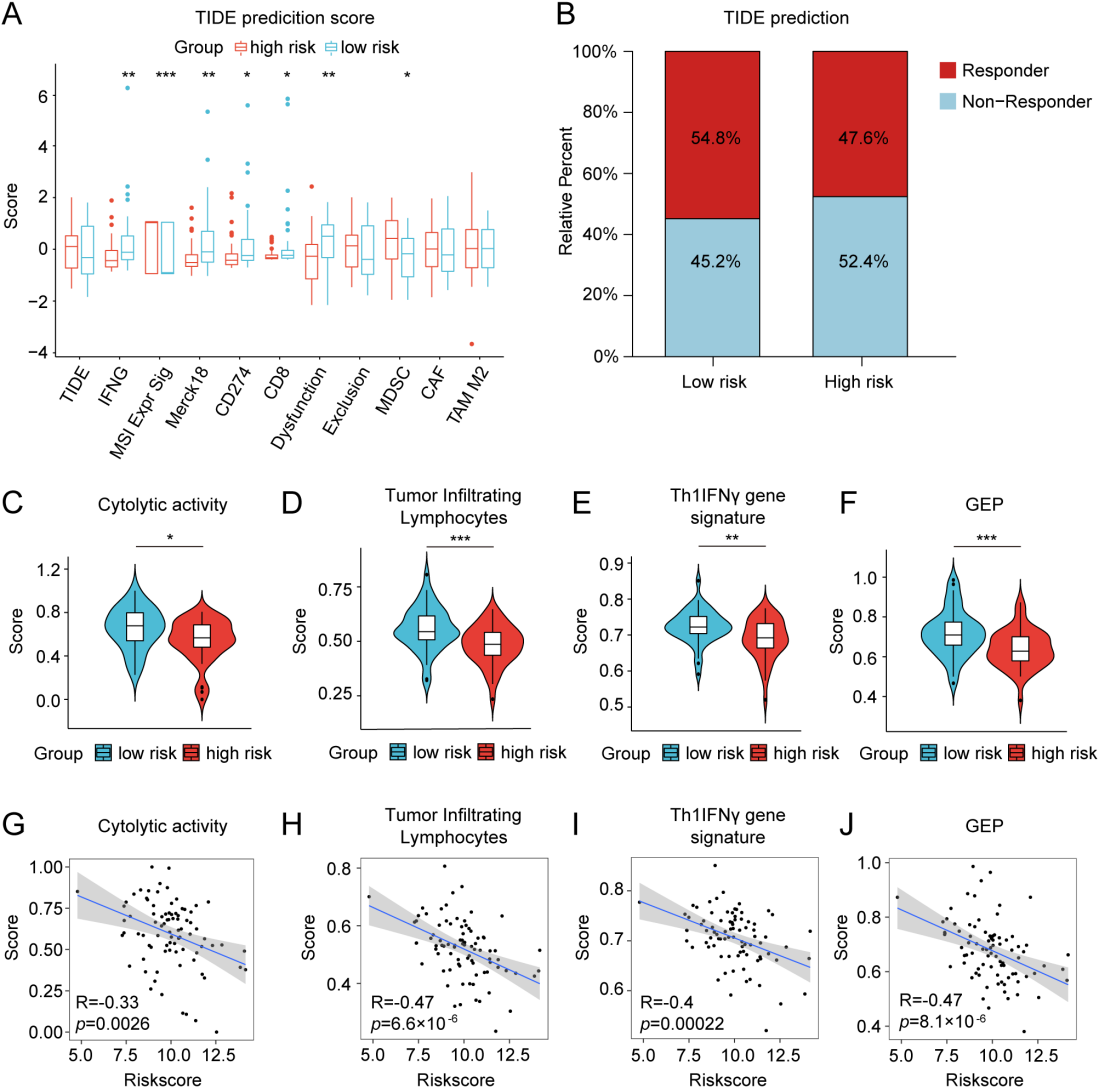
Differences in TIDE prediction and pathway activities of immunotherapy-related pathways between the low- and high-risk groups in the TARGET database. **(A)** TIDE prediction of the immunotherapy effectiveness of the two cohorts. **(B)** The bar graph depicting the proportion of patients sensitive and resistant to immunotherapy. **(C–F)** Violin plots presenting the distinctions of cytolytic activity score, tumor infiltrating lymphocyte score, Th1/IFN gene signature score, and T-cell-inflamed gene expression profile (GEP) score. **(G–J)** Correlation analysis between the risk score and the four scores mentioned above. ∗ *p* < 0.05, ∗∗ *p* < 0.01, ∗∗∗ *p* < 0.001.

**Figure 8:**
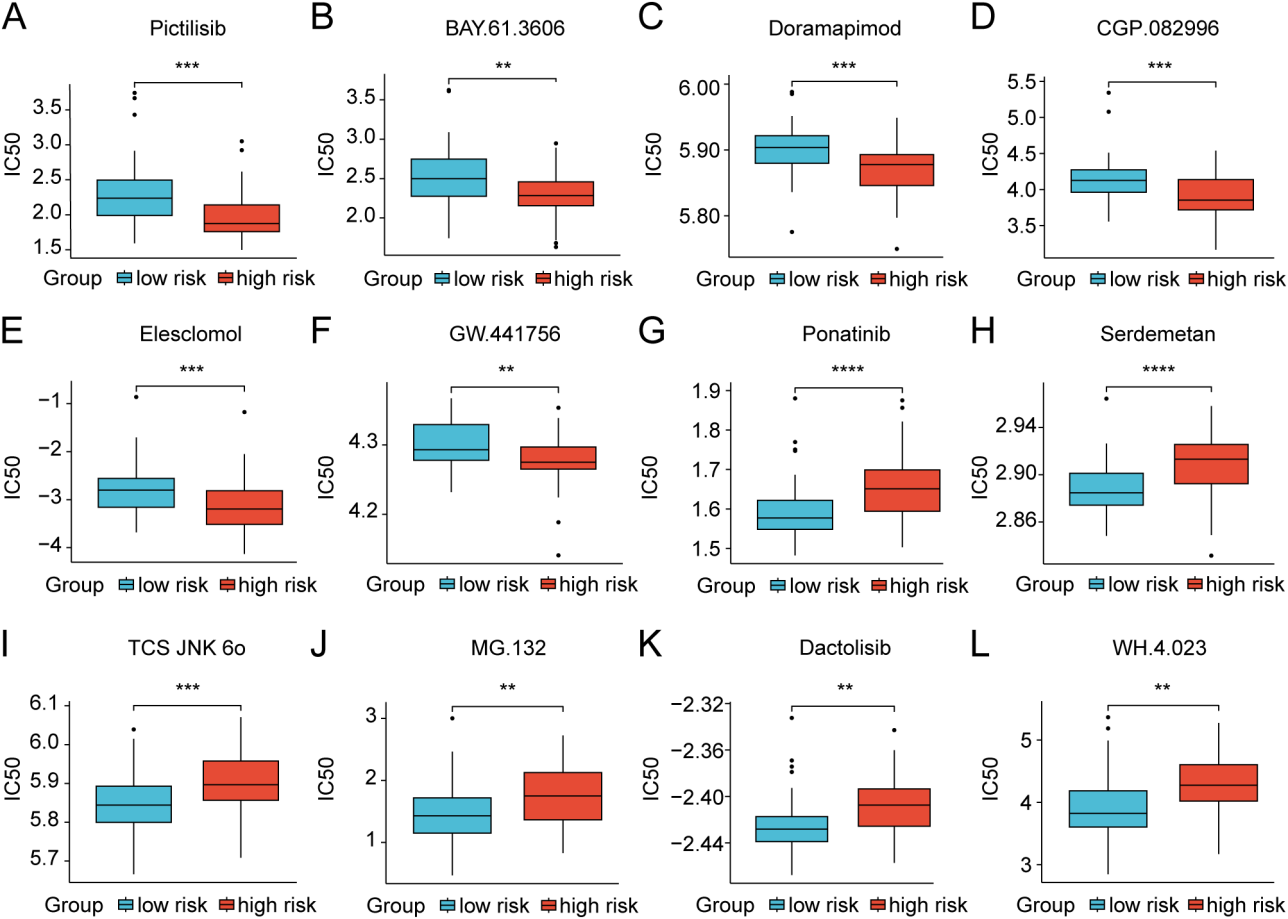
Discrepancies in treatment response to chemotherapy drugs of osteosarcoma patients between the low- and high-risk groups in the TARGET dataset. **(A–L)** Box plots showing the IC50 values for clinical drugs with statistical significance amidst the two risk groups. ** *p* < 0.01, *** *p* < 0.001, **** *p* < 0.0001.

We further investigated the differential expression of immune checkpoints, MHC molecules, and immunomodulators in low- and high-risk groups to predict the efficacy of immunotherapy in osteosarcoma samples. As essential prognostic markers of immunotherapy response, some immune checkpoints were considerably upregulated in the low-risk patients in the TARGET and GSE21257 cohorts, including *BTN3A1*, *LAG3*, *CD209*, *TNFSF4*, *BTN2A2*, *CD70*, *TNFSF14*, *HAVCR2*, *CD96*, *CD200R1*, *LGALS9*, *TIGIT*, *PDCD1LG2*, and *SIRPA* (**Figure 6A** and **Supplementary Figure S7A**). Additionally, a remarkably negative correlation was observed between the risk scores and the expression levels of these immune checkpoints (**Figure 6B**). Furthermore, a majority of MHC molecules, such as *HLA-DQA1*, *HLA-DOA*, *HLA-DMA*, *HLA-DRA*, *HLA-E*, *HLA-DPB1*, *HLA-DPA1*, and *HLA-DMB*, were expressed at considerably high levels in the low-risk group (**Figure 6C** and **Supplementary Figure S7B**). Meanwhile, the levels of most chemokines, interleukins, interferons, receptors, and other cytokines were also increased in the low-risk group, implying the enhanced sensitivity to immunomodulators and better response to immunotherapy compared to those of high-risk group (**Figure 6D**). The IFNG scores, Merck18 scores and T-cell dysfunction scores were found to be higher in the low-risk group of the TARGET and GSE21257 cohorts, while MDSC scores were lower in this group according to data from the TIDE website (**Figure 7A**). Remarkably, TAM M2 scores were increased in the high-risk group of the GSE21257 cohort (**Supplementary Figure S7C**). Subsequent analysis of immunotherapy response from the TIDE website exhibited a higher percentage of responders in the low-risk group (54.8% *vs.* 47.6%), suggesting that high-risk patients may have a tendency to escape immune surveillance and exhibit a less favorable response to immune checkpoint inhibitors (ICIs) (**Figure 7B**). Additionally, the T-cell-inflamed gene expression profile (GEP) score, Th1/IFN gene signature score, cytolytic activity score, and tumor infiltrating lymphocyte score were significantly elevated in the low-risk samples (**Figures 7C–F** and **Supplementary Figures S7D–G**) and displayed a negative correlation with risk scores (**Figures 7G–J**). The assessment of chemotherapy response through the computation of half-maximal inhibitory concentration (IC50) values for more than 100 chemotherapeutic agents indicated that high-risk patients were more likely to benefit from pictilisib, BAY.61.3606, doramapimod, CGP.082996, Elesclomol, and GW.441756, while the low-risk group was predicted to exhibit greater sensitivity to ponatinib, serdemetan, TCS JNK 6o, MG.132, dactolisib, and WH.4.023 (**Figures 8A–L**). These findings disclosed that the efficacy of immunotherapy and chemotherapy differed in the HLMRGS-based groups, and low-risk patients tended to have improved immunotherapy outcomes.

### 3.7 Single-cell analysis of the HLMRGs in TME-associated cells

**Figure 9:**
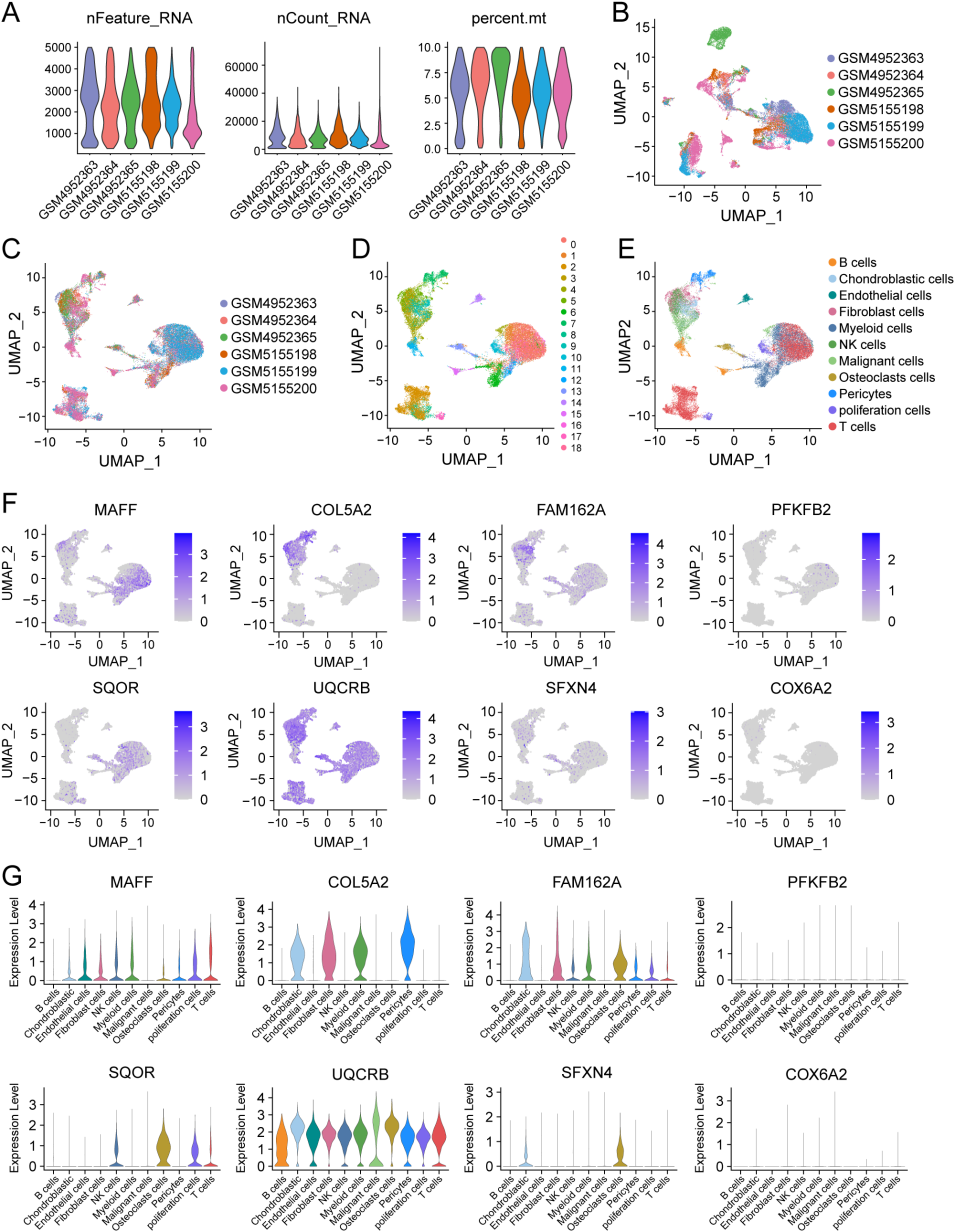
Single-cell analysis of 8 model genes in the GSE162454 dataset. **(A–C)** Quality control and de-batching effect of the single-cell sequencing raw data. **(D)** The UMAP plot of 30,660 cells colored by the 19 clusters. **(E)** The UMAP plot of 30,660 cells colored by 11 different cell subtypes. **(F, G)** The expression of 8 modeled genes across various cell subtypes.

Considering the tight association of the risk scores with TME, we examined the expression of 8 HLMRGs in various TME-associated cells at the single-cell level using the GSE162454 dataset. Following the implementation of initial quality control procedures, a gene expression profile was generated for a total of 30,660 cells encompassing 33,538 genes obtained from the primary tumors of 6 osteosarcoma samples (**Figures 9A, B**). The Harmony package was employed to mitigate batch effects (**Figure 9C**). Through unbiased clustering of the cells, 19 main clusters were identified in parallel, based on uniform manifold approximation and projection (UMAP) analyses of their gene profiles (**Figure 9D**). Subsequent cell annotation was performed using markers specific to different cell types as described in a previously published study, identifying 11 distinct cell populations denoted as B cells, chondroblastic cells, endothelial cells, myeloid cells, NK cells, osteoblastic cells, malignant cells, T cells, pericytes, proliferation cells, and fibroblast cells (**Figure 9E**). In general, *UQCRB* was found to be the most highly expressed gene, while *COX6A2* exhibited the lowest expression across all TME-related cell subtypes (**Figure 9F**). Remarkably, *SQOR* displayed significant variation in gene expression between malignant cells and osteoclasts (**Figures 9F, G**). The tumor suppressor genes *PFKFB2* showed quite low expression in malignant cells, which was consistent with its role as a protective factor in osteosarcoma as predicted by bioinformatics.

### 3.8 Experimental validation of the expression and biological functions of *SFXN4* and *SQOR*

**Figure 10:**
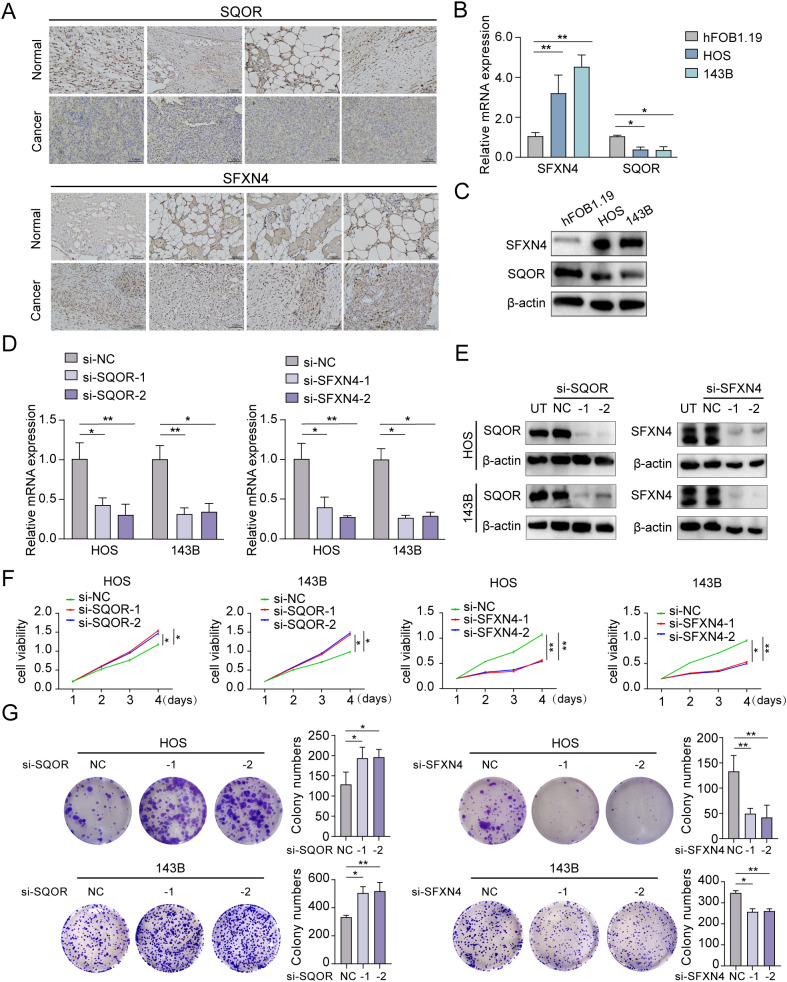
Expression and biological functions on growth of *SFXN4* and *SQOR* in osteosarcoma. **(A)** *SFXN4* and *SQOR* expression in para-cancerous and osteosarcoma tissues by IHC. **(B, C)** The expression of *SFXN4* and *SQOR* at RNA and protein levels in normal osteoblast cell and osteosarcoma cell lines through qRT-PCR and western blotting assays. **(D, E)** Effect of knocking down *SFXN4* and *SQOR* at RNA and protein levels in osteosarcoma cell lines by qRT-PCR and western blotting assays. **(F, G)** CCK8 and colony formation assays demonstrating the proliferation capacity ability after knocking down *SFXN4* and *SQOR* in osteosarcoma cell lines. ∗ *p* < 0.05, ∗∗ *p* < 0.01.

**Figure 11:**
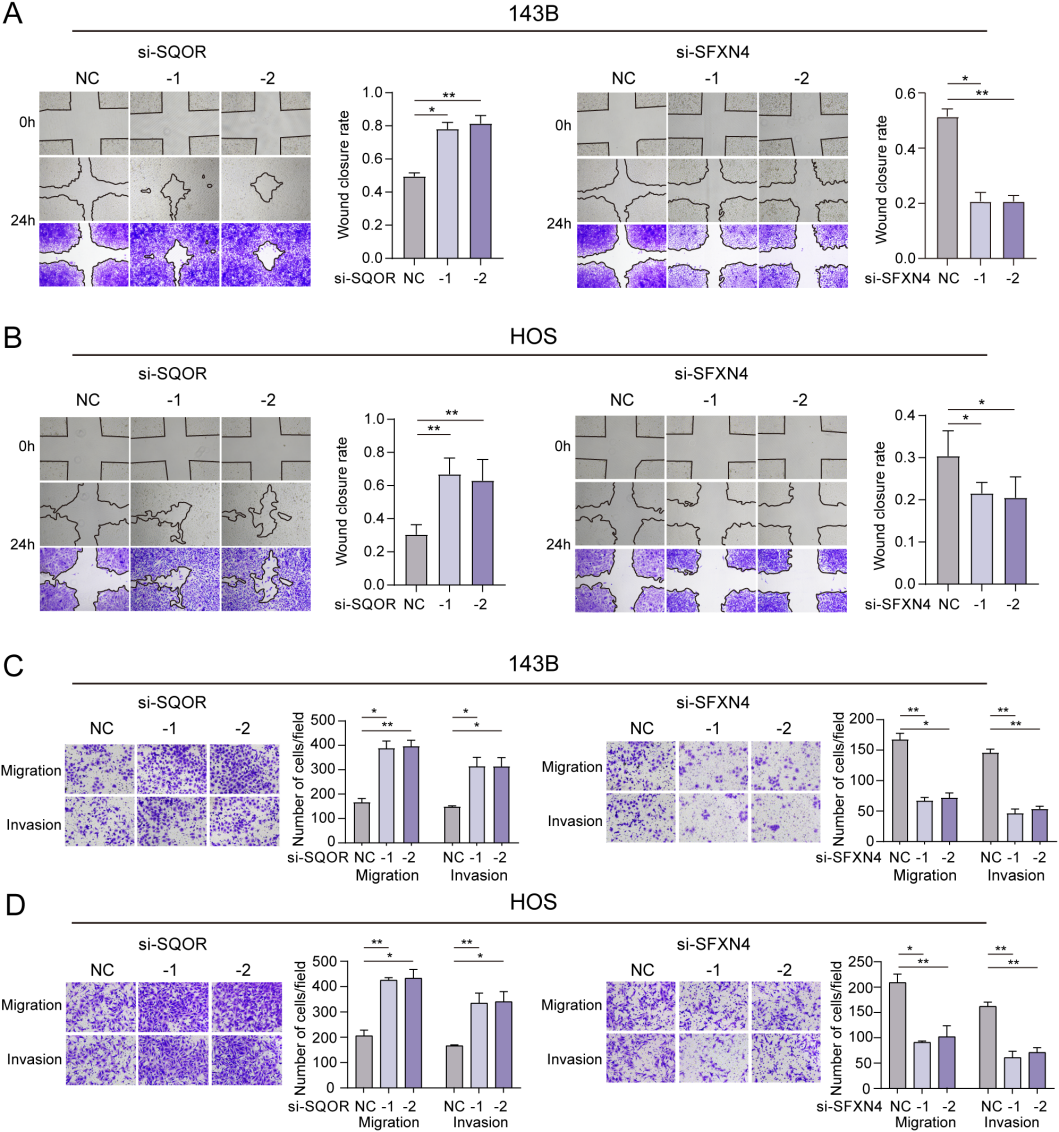
Effect of *SFXN4* and *SQOR* on invasion and metastasis of osteosarcoma. **(A, B)** Wound healing assay revealing the impact of *SFXN4* and *SQOR* knockdown on osteosarcoma cell invasiveness. **(C, D)** The invasive and migratory abilities of HOS and 143B cell lines were assessed by Transwell assays after *SFXN4* and *SQOR* knockdown. ∗ *p* < 0.05, ∗∗ *p* < 0.01.

**Figure 12:**
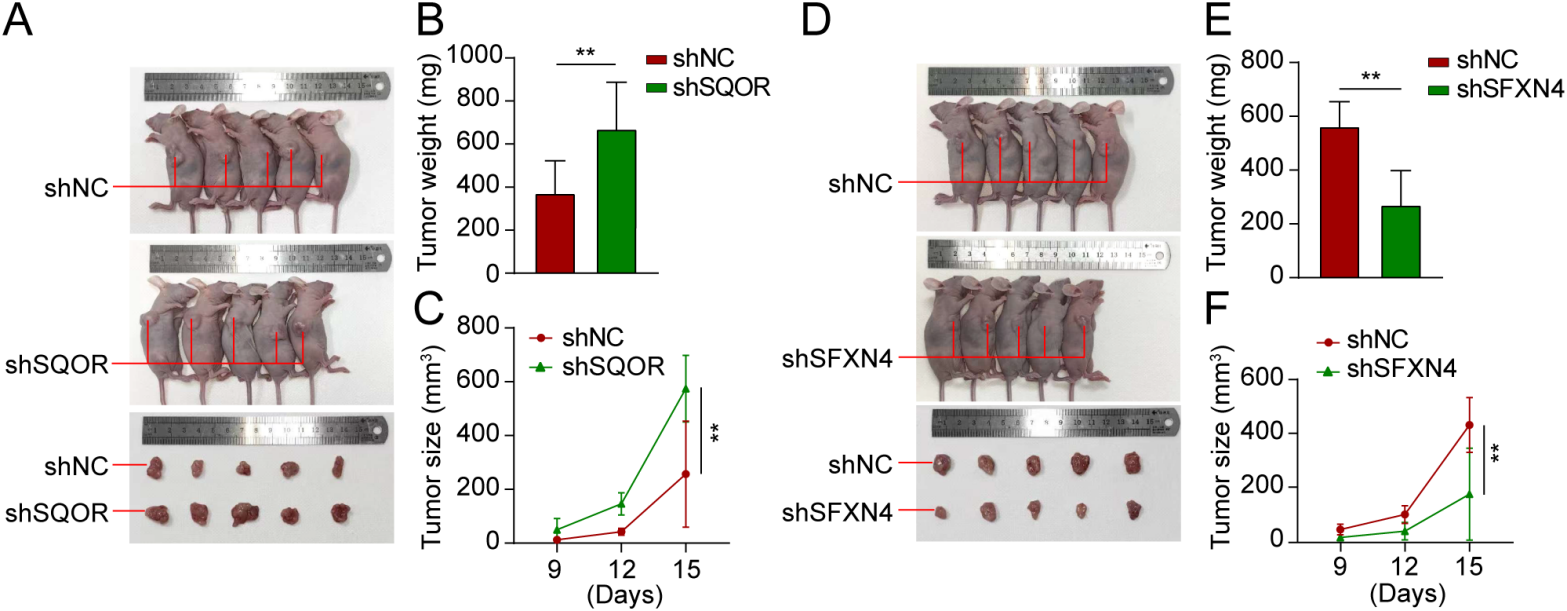
Knockdown of *SFXN4* and *SQOR* affected osteosarcoma growth *in vivo*. 143B cells were transfected with lentivirus expressing either shNC, sh*SFXN4*, or sh*SQOR*. **(A)** Knockdown of *SFXN4* significantly inhibited tumor growth. **(B, C)** Volume changes and weight of shNC and sh*SFXN4* tumors. **(D)** Knockdown of *SQOR* promoted tumor growth. **(E, F)** Volume changes and weight of shNC and sh*SQOR* tumors. **p < 0.01.

We selected two genes that have not been reported in osteosarcoma for further experimental validation. These genes were chosen based on their classification as protective and hazardous factors, respectively (**Figure 2E**). *SQOR* was picked out from two protective elements in the multivariate Cox regression on account of its visibly low *p* value. Additionally, the decision to focus on *SFXN4* was supported by its obviously high HR value compared to other modeled genes. The RT-qPCR, western blot and immunohistochemistry (IHC) analyses confirmed that *SFXN4* displayed elevated expression at both RNA and protein levels in osteosarcoma tissues and cells, whereas *SQOR* exhibited heightened abundance in normal tissues and cells (**Figures 10A–C**). Transfection of short interfering RNA fragments into HOS and 143B cell lines resulted in decreased expression levels of *SFXN4* and *SQOR* (**Figures 10D, E**). Knockdown of *SFXN4* led to decreased cell proliferation, migration, and invasion, while knockdown of *SQOR* had the opposite effect (**Figures 10F, G**, **11A–D**). Then, we performed *in vivo* experiments to further investigate the functions of *SFXN4* and *SQOR* in osteosarcoma. As shown in **Figures 12A–C**, knockdown of *SQOR* enhanced tumor growth, whereas knockdown of *SFXN4* yielded an opposing effect (**Figures 12D–F**).

## 4 Discussion

Hypoxia and lactate metabolism are two widely recognized characteristics of the tumor microenvironment (TME) that profoundly affect tumor progression, immune infiltration, and treatment response (33–38). This study has introduced a novel gene signature related to hypoxia and lactate metabolism (HLMRGS), representing the first identification of such a signature in osteosarcoma. Moreover, the research also underscored the clinical relevance of the HLMRGS concentrated on its latent force for predicting prognosis and immunotherapy response.

Previous studies primarily stressed on gene signatures related to hypoxia or lactate metabolism in osteosarcoma (39, 40). However, a gene signature that integrates both hypoxia and lactate metabolism for prognostic evaluation and treatment guidance in osteosarcoma has not yet been reported. The area under the curve (AUC) values of the HLMRGS surpassed those of existing models, indicating its superior predictive performance. Besides, compared to other models related to hypoxia or lactate metabolism, our AUC values stood out as the highest, showcasing exceptional predictive capability (41–47). This suggested that the HLMRGS was well suitable for osteosarcoma patients and served as a valuable tool for predicting clinical outcomes.

Immune cells are the cellular foundation of immunotherapy (48). The abundance and variety of tumor-infiltrating immune cells are intricately linked to therapeutic efficacy prediction and clinical outcomes (49). The immune cell composition of individuals with the same cancer may vary significantly inside the tumor microenvironment (TME), emphasizing the importance of characterizing immune infiltrates and their functional status for enhancing response rates (50). In our study, we observed that lower risk scores were closely correlated with more infiltration of crucial antitumor cells, including NK cells, CD4+ T cells, CD8+ T cells, cytotoxic T cells, and dendritic cells (DCs) within the TME. The cytotoxic activity of CD8+ T cells is a potent effector mechanism in tumor destruction (51), while CD4+ T cells are pivotal in initiating and coordinating innate and antigen-specific immune response (52). NK cells can recruit DCs to tumors to strengthen CD8+ T-cell response and improve the efficacy of immunotherapy by cooperating with T cells based on their complementary functions in tumor immunity (53). Besides, the low-risk group exhibited increased immune scores according to the ESTIMATE algorithm, indicating a more favorable TME enriched with abundant immune cells. It has been reported that osteosarcoma can be classified as an immune “hot” tumor or “cold” tumor or somewhere in between (54). Generally, “hot” tumors demonstrate higher response rates to immunotherapy (55). In our research, the tumors of low-risk patients were more likely to be classified as “hot” tumors more sensitive to immunotherapy due to more infiltration of crucial anti-tumor cells.

As a prevailing immunotherapy, immune checkpoint inhibitors (ICIs) treatment takes into account the genetic background of tumors by utilizing biomarker-based patient selection, thereby hopefully increasing the proportion of osteosarcoma patients who benefit from the ICIs treatment (5). In our study, we observed that the subset classified as “hot” exhibited an adaptive immune resistance mechanism characterized by the upregulation of T cell inhibitory immune-checkpoint proteins, specifically *TIGIT* (32). Besides, *PDCD1LG2* blockade may become potential immunotherapeutic intervention targets enhancing the cytotoxic effects against osteosarcoma (56). Overall, low-risk patients were more likely to be categorized as individuals with “hot” tumors and harbored a better prognosis after ICIs treatment thanks to more infiltration of tumor-killing cells with reversible dysfunction.

Additionally, low-risk patients exhibited increased expression levels of most HLA genes, interleukin-2 (IL-2), and heightened activity in the M2-like tumor-associated macrophages (TAMs) pathway, as well as an elevated gene expression profile (GEP) score. The majority of “cold” tumors displayed decreased expression levels of HLA antigen-presenting genes, leading to the absence of TCR productive clonality and subsequent immune evasion (57). The combination of IL-2 and chemotherapy demonstrate a three-year survival rate exceeding 40% among patients (58). TAMs may create an immunosuppressive environment to participate in the malignant progression of osteosarcoma (59). As a novel gene signature biomarker for patient's response to ICIs, a higher GEP score implies worse treatment effects (60). These results were in line with the findings of the TIDE prediction analysis, indicating that low-risk individuals may derive greater benefits from immunotherapy interventions.

Most modeled genes included in the HLMRGS have been demonstrated to be connected to tumor microenvironment or immunotherapy efficiency across various cancers in prior studies. Here, our bioinformatic correlation analysis suggested that *SQOR* was positively correlated with the infiltration abundance of nearly all immune cells, with particularly strong correlations observed for NK T cell and Gamma delta T cell infiltration. NK T cells are a specialized subpopulation of T cells that expressed both T cell receptor and NK cell receptor (61). Beyond their direct cytotoxic effects on cancer cells, they also modulate the immune response by secreting a large number of cytokines and chemokines (e.g., IL-4, IFN-V, etc.) (62). Gamma delta T cells are recognized as the most tumor-killing immune cell; they not only induce apoptosis in tumor cells but also activate NK cells through 4-1BBL expression (63). Hence, we hypothesized that *SQOR* may activate the interaction network between NK T cells and Gamma belta T cells, thereby increasing the abundance of immune cell infiltration, altering the tumor microenvironment, and enhancing the patient’s response to immunotherapy. *SFXN4* has been shown to be associated with multimodal immune cell infiltration in hepatocellular carcinoma (64). Unfortunately, our analysis showed that this correlation did not hold true in osteosarcoma. Nevertheless, the results of our *in vivo* and *in vitro* experiments supported a critical role of *SFXN4* in tumor progression. However, the molecular mechanism of *SFXN4* in the malignant progression of osteosarcoma needs to be further investigated. *COL5A2* was found to enhance the infiltration abundance of tumor-associated macrophages while concurrently diminishing the population of CD8 T cells in prostate cancer (65). *FAM162A* has been incorporated into the prediction model of lung squamous cell carcinoma, and its expression has been taken into account for calculating the m6A score to predict the immunotherapy response of patients (66). *UQCRB* can ensure the normal function of mitochondrial complex III and rectify hypoglycemia and lactic acidosis in the tumor microenvironment of colorectal cancer (67). *COX6A2* was reported to be incorporated into the oxidative phosphorylation gene model of osteosarcoma for predicting the prognosis of patients and the infiltration of immune cells (68). Last but not the least, we conducted *in vivo* experiments to investigate the effects of *SQOR* and *SFXN4* on the growth of osteosarcoma. The results demonstrated that *SQOR* exhibited a tumor-suppressive function, whereas *SFXN4* facilitated tumor growth. These findings aligned with our bioinformatics predictions, indicating that both *SQOR* and *SFXN4* may serve as potential drug targets for future clinical treatment of osteosarcoma. This opened new avenues for targeted therapy aimed at osteosarcoma patients. However, further research is required to determine whether *SQOR* and *SFXN4* influence the growth of osteosarcoma through the tumor microenvironment and tumor immunity.

Our study had several limitations. The signature was developed and validated through bioinformatics analyses using publicly available databases. Although we have assessed its reliability in external validation sets, a large-scale clinical trial is necessary for further validation. Moreover, additional research on the efficacy of immunotherapy and drug sensitivity assays is needed for clinical evaluation in future studies. Finally, the specific molecular mechanisms by which *SQOR* and *SFXN4* affect the tumor microenvironment and immunotherapeutic response need to be further investigated.

## 5 Conclusions

In summary, this study developed and validated a powerful hypoxia- and lactate metabolism-related gene signature that effectively predicted patient’s prognosis and response to immunotherapy. *In vivo* and *in vitro* experiments demonstrated that the expression of *SQOR* could suppress the growth of osteosarcoma, whereas the expression of *SFXN4* promoted it. Additionally, *in vitro* experiments also verified that *SQOR* inhibited the metastasis and invasiveness of osteosarcoma cells, while *SFXN4* had the opposite effect. These findings will contribute to understanding the tumor microenvironment in osteosarcoma and guiding the selection of immune checkpoint inhibitors in osteosarcoma treatment strategies, thus improving individualized therapy and enlarging the population benefiting from immunotherapy.

## Data Availability

The original contributions presented in the study were included in the article/**Supplementary Material**. Further inquiries can be directed to the corresponding author.
