# Supplementary material for: A novel hypoxia- and lactate metabolism-related prognostic signature to characterize the immune landscape and predict immunotherapy response in osteosarcoma
Source: Front Immunol. 2024 Nov 6;15:1467052. doi: 10.3389/fimmu.2024.1467052 (PMC11576178; doi:10.3389/fimmu.2024.1467052)
Supplement: Supplementary file 2 [file DataSheet1.docx]

**A novel hypoxia- and lactate metabolism-related prognostic signature to characterize the immune landscape and predict immunotherapy responses in osteosarcoma.**

**Yizhuo Wang^1^,** **Yang Liu^1^,** **Jiayuan Xu^1^, Jiyuan Zhu^2^, Yufu Zheng^1^, Quan Qi^1*^**

*** Correspondence:**Quan Qi
qiquan@hrbmu.edu.cn.

Supplementary Material

**
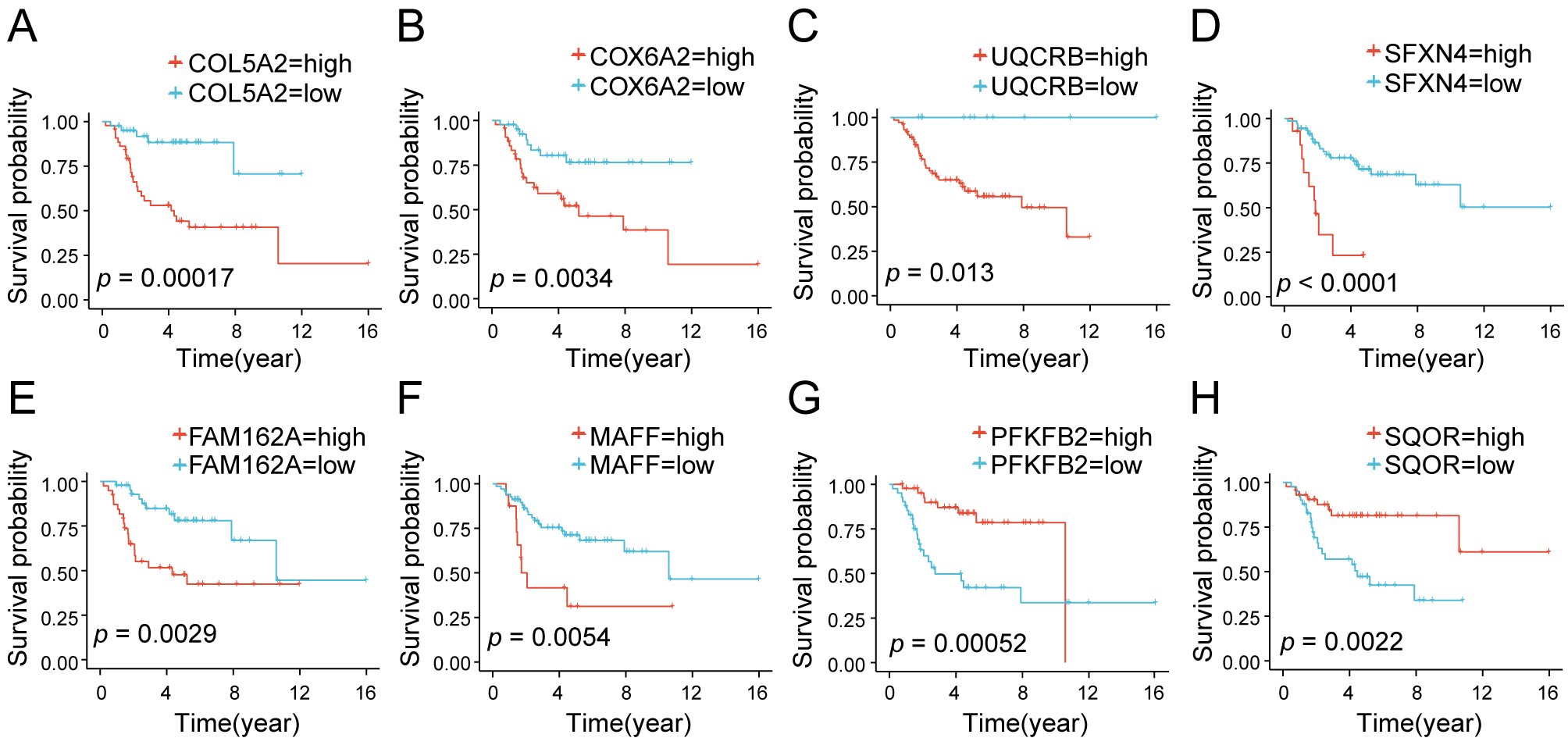
**

**Supplementary Figure 1**. KM survival analyses of 8 modeled genes in the TARGET cohort. (A-H) KM survival analysis of 8 modeled genes, including *MAFF*, *COL5A2*, *FAM162A*, *SQOR*, *UQCRB*, *SFXN4*, *PFKFB2* and *COX6A2*.

**
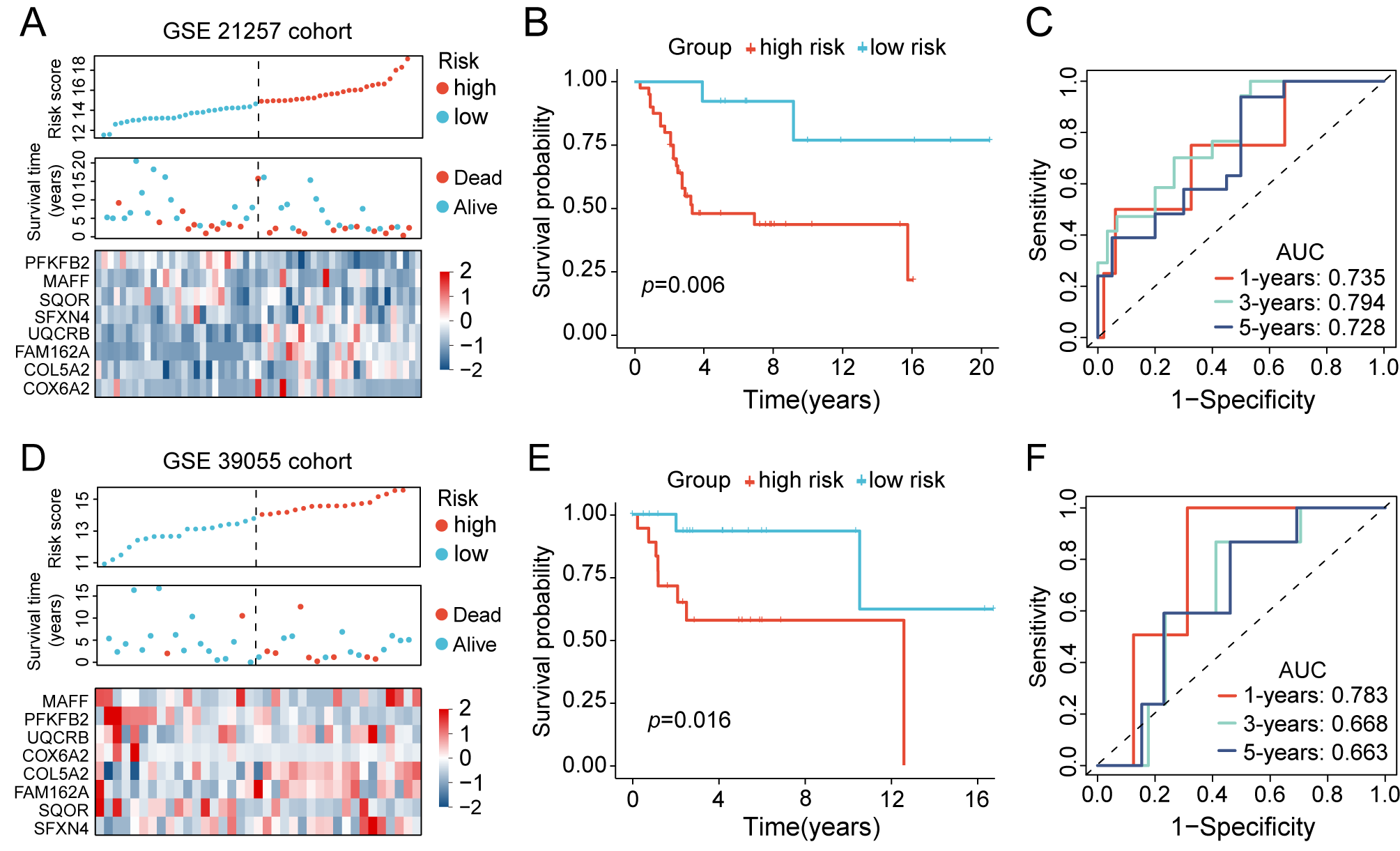
**

**Supplementary Figure 2.** Verification using the HLMRGS to predict patient outcomes in other validation datasets GSE21257 and GSE39055. (A, D) The risk scores, clinical outcomes, and expression of 8 modeled genes of osteosarcoma samples divided by median risk score in two datasets. (B, E) KM survival analysis for the low- and high-risk groups in two datasets. (C, F) The ROC curve showing the predictive potential of HLMRGS in two datasets.

**
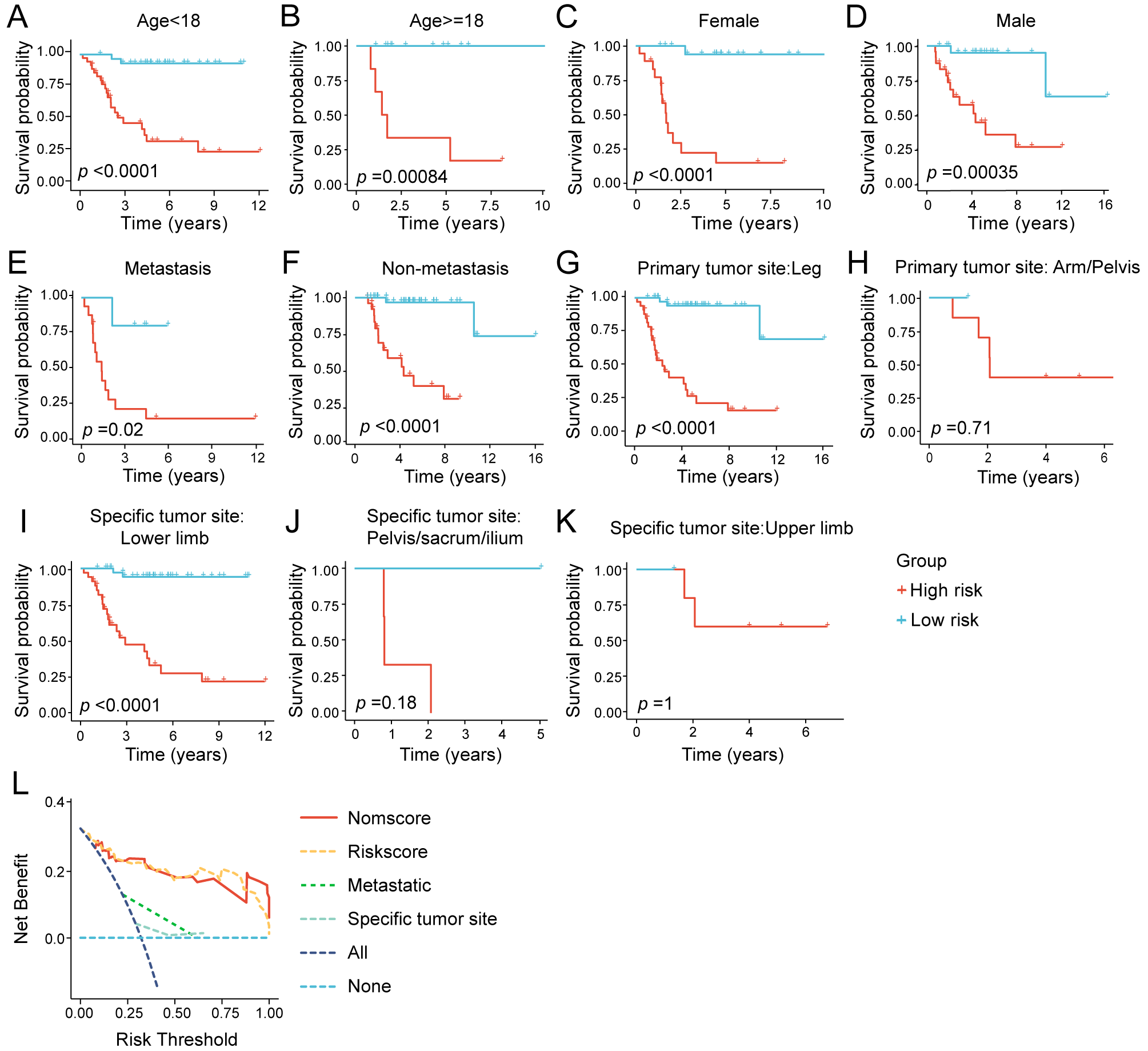
**

**Supplementary Figure 3.** KM survival analysis amidst the low- and high-risk groups in disparate subgroups divided by various clinical features in the TARGET cohort. KM survival analysis of different subgroups containing age [<18 years vs. >=18 years] (A, B), sex [female vs. male] (C, D), metastasis [metastatic vs. nonmetastatic] (E, F), primary tumor site [leg vs. arm/pelvis] (G, H), and specific tumor site [lower limb vs. pelvis/sacrum/ilium vs. upper limb] (I-K). (L) DCA plot showing the net survival benefits of HLMRSG and other clinical indications.

**
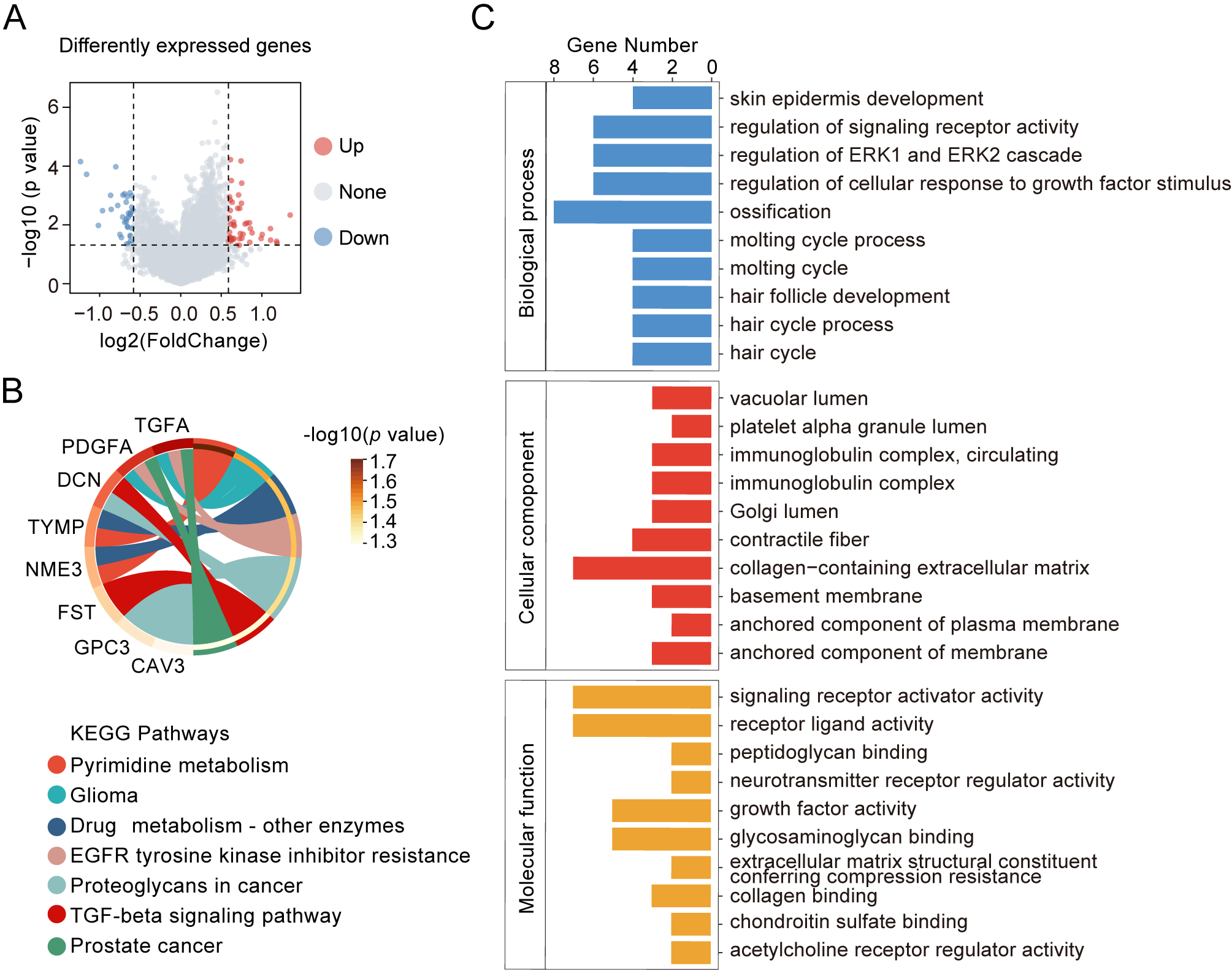
**

**Supplementary Figure 4.** Functional enrichment analysis of the low- and high-risk groups in the TARGET database. (A) A volcano map of the DEGs between the two cohorts. (B) Enrichment circle displaying the association between key DEGs and enrichment pathways involved in the KEGG. (C) Bar charts showing GO enrichment analyses from three aspects.

**
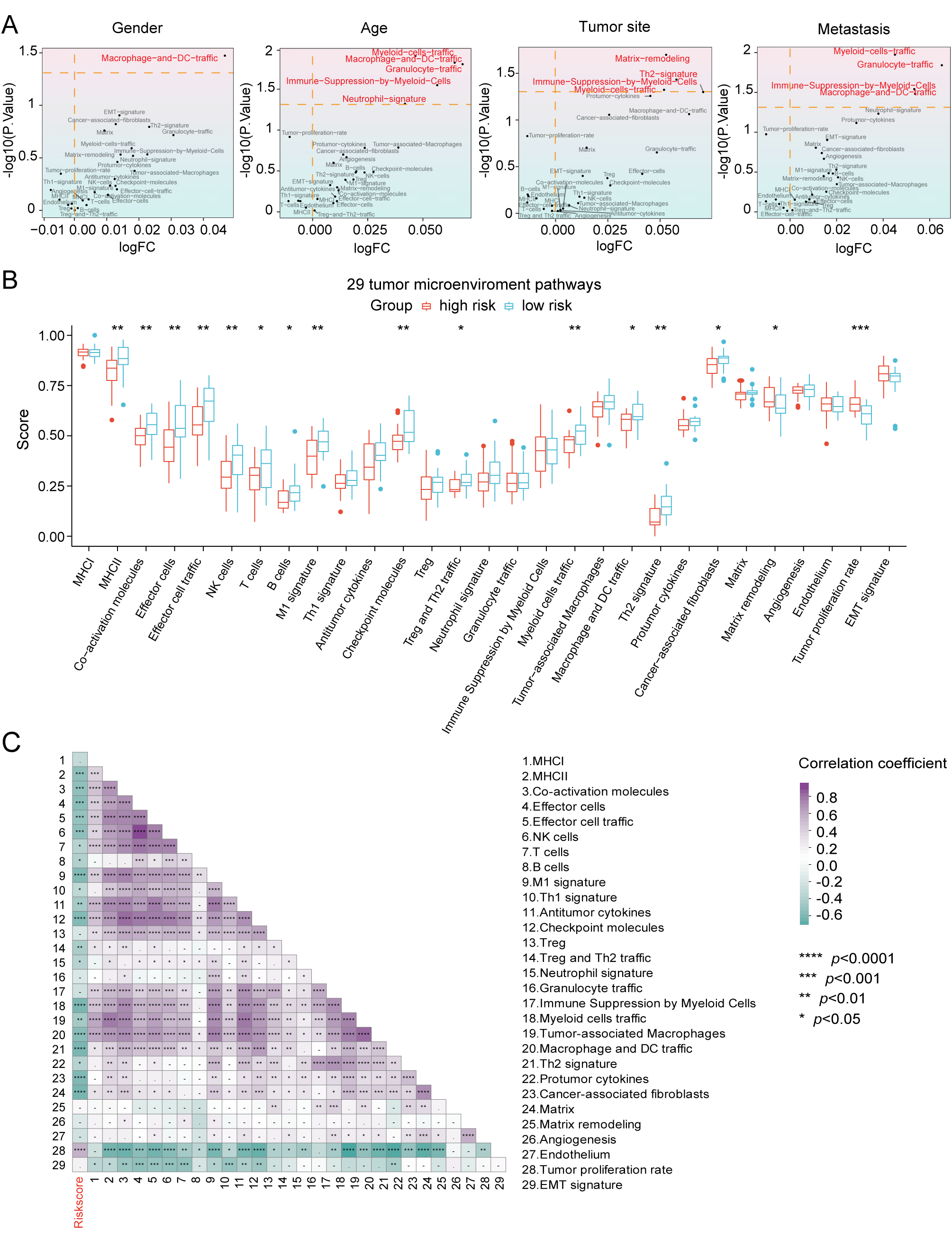
**

**Supplementary Figure 5.** Heterogeneity of the tumor microenvironment amidst the low- and high-risk groups in the GSE21257 dataset. (A) The differentially expressed activity of 29 TME pathways between the two subgroups categorized on sex, age, tumor location, and metastasis by using “limma” package. (B) Box line plot depicting the activity discrepancies of 29 TME pathways between the two groups. (C) Multidimensional correlation plot showing the correlation between risk scores and 29 TME pathways. Using a Wilcoxon rank-sum test, the significance level was determined (ns *p* > 0.05, ∗ *p* < 0.05, ∗∗ *p* < 0.01, ∗∗∗ *p* < 0.001, ∗∗∗∗ *p* < 0.0001).

**
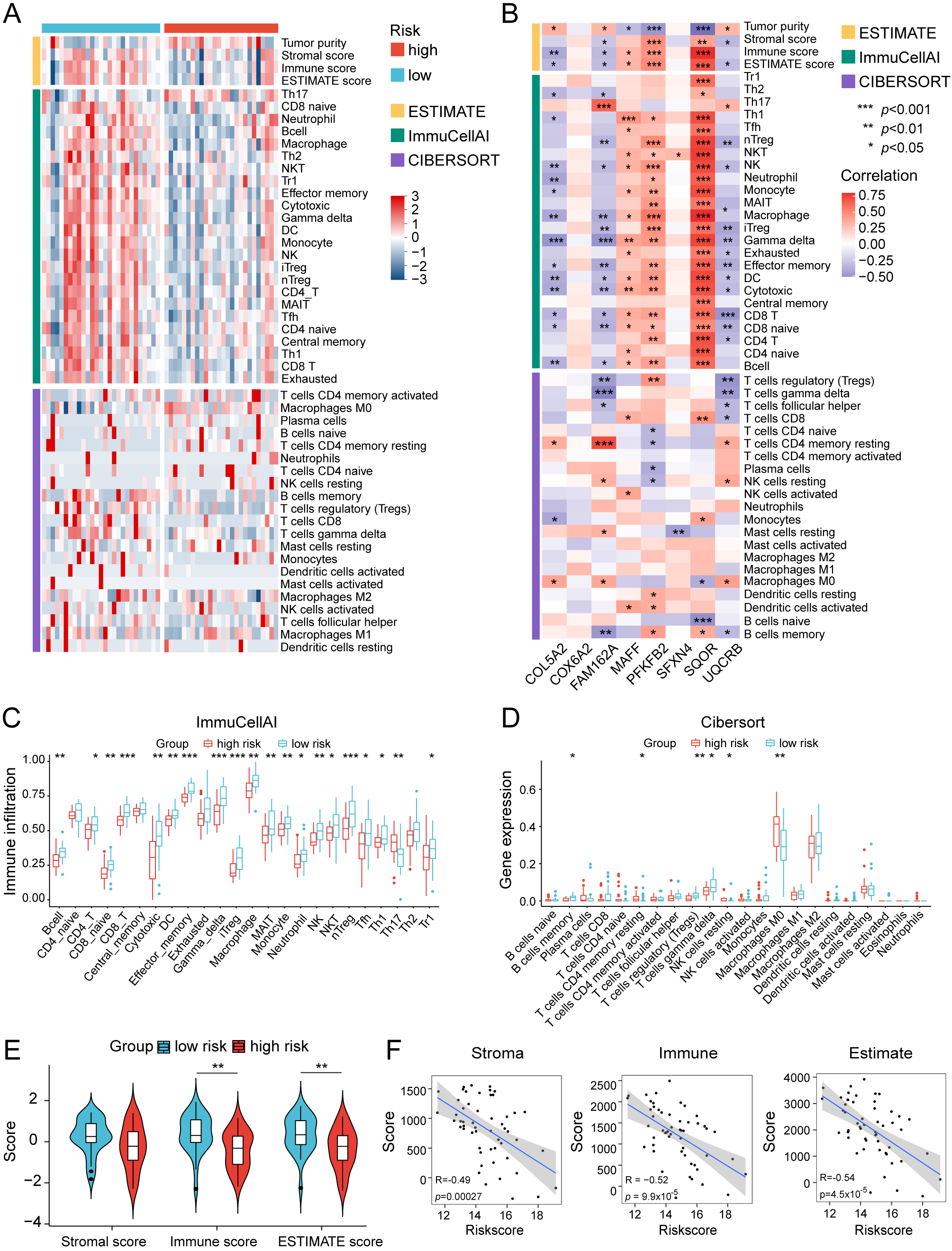
**

**Supplementary Figure 6.** Divergences in the immune environment between the low- and high-risk groups in the GSE21257 dataset. (A) Heatmap showing the variances in immune infiltration with the ESTIMATE, ImmuCellAI, and CIBERSORT algorithms between the two cohorts. (B) Correlation plot depicting the correlation between the expression of modeled genes and ESTIMATE, ImmuCellAI, and CIBERSORT scores. (C) Infiltration diversity of 24 immune cell subpopulations based on the ImmuCellAI database between the two groups. (D) Infiltration discrepancies of 22 immune cell subpopulations with the CIBERSORT algorithm. (E) Distinctions of stromal, immune, and ESTIMATE scores between the two risk groups. (F) Correlation analysis of risk scores with stromal, immune, and ESTIMATE (ns *p* > 0.05, ∗ *p* < 0.05, ∗∗ *p* < 0.01, ∗∗∗ *p* < 0.001, ∗∗∗∗ *p* < 0.0001).


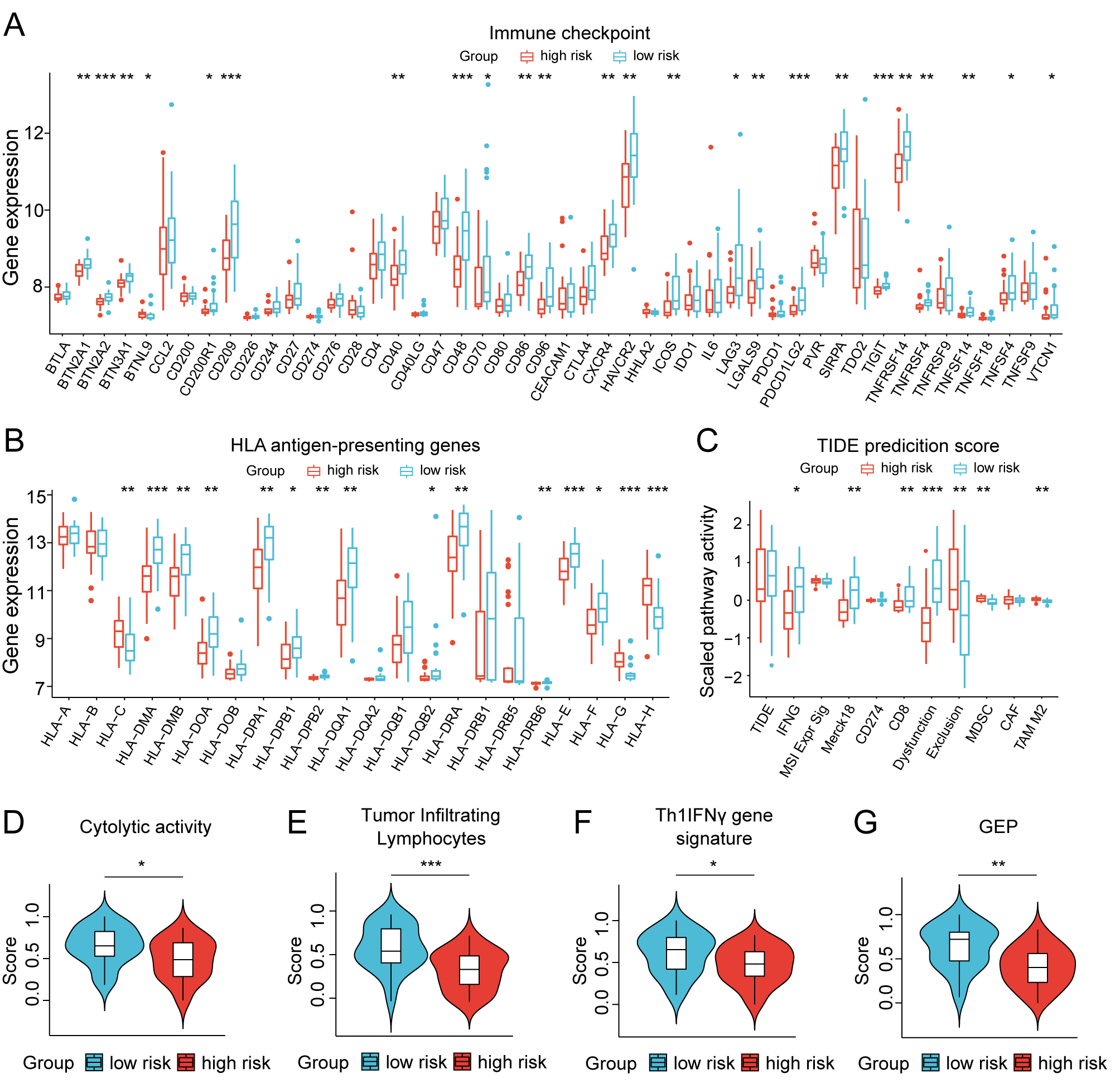


**Supplementary Figure 7.** Assessment of immunotherapy efficacies in the low- and high-risk groups of the GSE21257 cohort. (A) Box plot showing the distinctions in the gene expression of immune checkpoints between the two risk groups. (B) Discrepancies in the expression of MHC molecules between the two cohorts. (C) TIDE prediction of the immunotherapy effectiveness of the low- and high-risk groups. (D-G) Violin plots illustrating the differences in cytolytic activity score, tumor infiltrating lymphocyte score, Th1/IFN gene signature score, and T-cell-inflamed gene expression profile (GEP) score between the two groups (ns *p* > 0.05, ∗ *p* < 0.05, ∗∗ *p* < 0.01, ∗∗∗ *p* < 0.001, ∗∗∗∗ *p* < 0.0001).
